# Transcription Factors MYOCD, SRF, Mesp1 and SMARCD3 Enhance the Cardio-Inducing Effect of GATA4, TBX5, and MEF2C during Direct Cellular Reprogramming

**DOI:** 10.1371/journal.pone.0063577

**Published:** 2013-05-21

**Authors:** Nicolas Christoforou, Malathi Chellappan, Andrew F. Adler, Robert D. Kirkton, Tianyi Wu, Russell C. Addis, Nenad Bursac, Kam W. Leong

**Affiliations:** 1 Department of Biomedical Engineering, Duke University, Durham, North Carolina, United States of America; 2 University of Texas Southwestern Medical School, Dallas, Texas, United States of America; 3 Institute for Regenerative Medicine and Department of Cell and Developmental Biology, Perelman School of Medicine, University of Pennsylvania, Philadelphia, Pennsylvania, United States of America; University of Tampere, Finland

## Abstract

Transient overexpression of defined combinations of master regulator genes can effectively induce cellular reprogramming: the acquisition of an alternative predicted phenotype from a differentiated cell lineage. This can be of particular importance in cardiac regenerative medicine wherein the heart lacks the capacity to heal itself, but simultaneously contains a large pool of fibroblasts. In this study we determined the cardio-inducing capacity of ten transcription factors to actuate cellular reprogramming of mouse embryonic fibroblasts into cardiomyocyte-like cells. Overexpression of transcription factors MYOCD and SRF alone or in conjunction with Mesp1 and SMARCD3 enhanced the basal but necessary cardio-inducing effect of the previously reported GATA4, TBX5, and MEF2C. In particular, combinations of five or seven transcription factors enhanced the activation of cardiac reporter vectors, and induced an upregulation of cardiac-specific genes. Global gene expression analysis also demonstrated a significantly greater cardio-inducing effect when the transcription factors MYOCD and SRF were used. Detection of cross-striated cells was highly dependent on the cell culture conditions and was enhanced by the addition of valproic acid and JAK inhibitor. Although we detected Ca^2+^ transient oscillations in the reprogrammed cells, we did not detect significant changes in resting membrane potential or spontaneously contracting cells. This study further elucidates the cardio-inducing effect of the transcriptional networks involved in cardiac cellular reprogramming, contributing to the ongoing rational design of a robust protocol required for cardiac regenerative therapies.

## Introduction

Cardiovascular disease and ultimately heart failure resulting from myocardial infarction are caused mainly due to the lack of a sufficient number of cardiomyocytes - the contractile engine of the myocardium. Specifically, the average human adult left ventricle is comprised of 4 billion cardiomyocytes, and the sudden loss of approximately 25% of these cells due to an infarction injury leads to their eventual replacement with non-contractile scar tissue ultimately causing heart failure [1]. Over the past two decades significant effort has been made both in the lab and the clinic to prevent or even reverse heart failure through cell replacement and regeneration of the infarcted myocardium [2]. To fulfill the potential of such a therapeutic approach, a formidable challenge is to have available a sufficient number of *de novo* cardiomyocytes that can improve cardiac functional output [3].

Pluripotent embryonic stem (ES) cells, which theoretically have an unlimited expansion potential, can readily differentiate into spontaneously contracting cardiomyocytes, resembling the nascent myocardium in both function and electrophysiological properties [4], [5]. Moreover, the discovery that transient overexpression of four transcription factors (TFs) is sufficient to epigenetically reprogram terminally differentiated somatic cells [6], [7] into induced pluripotent stem (iPS) cells, which closely resemble ES cells and hold amongst the rest the same cardiac differentiation capacity [8], [9] further revolutionized the field. This powerful methodology has also formed the foundation for a new scientific direction: “direct epigenetic cellular reprogramming” or “induced transdifferentiation” which can be generally defined as the acquisition of a distinct alternative cellular phenotype from a particular cell lineage [9]–[11].

In particular, Ieda et al. reported that simple overexpression of three genes (Gata4, Tbx5, and Mef2c) in neonatal cardiac and dermal mouse fibroblasts can result in induction of cardiomyocyte-like cells in vitro [10]. Unsurprisingly, the three TFs used to achieve cellular reprogramming are positioned at the core of the genetic regulatory networks that govern developmental cardiogenesis across many evolutionary layers [12]. Two recent studies have reported similar results using either cardiac TFs or microRNA molecules [13], [14], although a third study brought into question the capacity of Gata4, Tbx5, and Mef2c to achieve complete cellular reprogramming into cardiomyocytes [15]. Importantly, it has been recently demonstrated that intramyocardial viral delivery and overexpression of these cardiac TFs in the infarcted myocardium can induce *in situ* reprogramming of cardiac fibroblasts into cardiomyocytes, and improve the functional output of the heart [16], [17]. Although significant strides have been made in this nascent field of epigenetic cardiac reprogramming, many issues require further investigation including the effect of additional cardiac TFs, the effect of the induction culture conditions, and the phenotypic characteristics of the reprogrammed cells.

In this study we evaluated the capacity of ten TFs to successfully induce cardiac cellular reprogramming of mouse embryonic fibroblasts (MEFs): *Mm Mesp1* (*M_1_*), *Hs SMARCD3* (*S_3_*), *Hs MYOCD* (*M_D_*), *Hs SRF* (*S_F_*), *Hs NKX2-5* (*N_5_*), *Hs HAND1* (*H_1_*), *Hs HAND2* (*H_2_*), *Hs GATA4* (*G_4_*), *Hs TBX5* (*T_5_*), *Hs MEF2C* (*M_C_*). The TFs were distributed into four modules and various module combinations were used to transduce MEFs utilizing an inducible-expression lentivirus-based gene delivery system. We established a set of criteria of increasing stringency to determine the cardio-inducing effect and cellular reprogramming capacity of particular module combinations: 1) TF-induced binding and activation of cardiac-specific promoter elements, 2) Expression of endogenous cardiac-specific genes including those encoding for cardiac cytoskeletal proteins, cardiac transcription factors, calcium handling proteins and cardiomyocyte ion channel proteins, 3) Phenotypic cellular metamorphosis associated with cytoskeletal remodeling or reorganization, and in particular detection of a cross-striated cytoskeleton, and 4) Cardiomyocyte-like functional properties including resting membrane potential, intracellular calcium cycling, and spontaneous contractile activity. We determined that the modest cardiac reprogramming effect of *GATA4*, *TBX5*, and *MEF2C* could be enhanced with the addition of either *MYOCD* and *SRF* alone or in conjunction with *Mesp1* and *SMARCD3*. Although transcription factor overexpression produced a strong and significant cardio-inducing effect, as determined by global gene expression analysis, formation of sarcomeres required fine-tuning of the culture conditions. Finally, we readily detected calcium transient cycling in reprogrammed cells although no significant differences were recorded when measuring the resting membrane potential.

## Materials and Methods

### Cell Culture and Reprogramming Induction

MEFs (Millipore, PMEF-HL & PMEF-NL), mouse NIH3T3 (ATCC, CRL-1658), and human HEK293T (ATCC, CRL-11268) were maintained and passaged in standard growth medium containing FBS (10%, Atlanta Biologicals), DMEM.HG (Life Technologies, 11960044), Glutamax (1X, Life Technologies, 35050058), Non-Essential Amino Acids (1X, Life Technologies, 11140050), Sodium Pyruvate (1X, Life Technologies, 11360070), βMercaptoethanol, (1X, Life Technologies, 21985023), and Gentamicin (25 µg/ml, Lonza, 17-518Z). Cells were passaged on tissue culture polystyrene pre-coated with porcine skin gelatin (Sigma, G1890). Transgenic MEFs isolated from mouse embryos (13 d.p.c.), were expanded in standard growth media conditions. To generate the transgenic animals the Myh6 promoter element was used to drive expression of eGFP and Puromycin N-acetyl-transferase (PAC). The transgenic mice were maintained in a mixed 129Svev/C57BL/6 background. For the isolation of primary mouse embryonic fibroblasts, a single pregnant female mouse was euthanized by CO2 asphyxiation. Animal procedures were conducted according to the guidelines for the care and use of laboratory animals set and approved by the Institutional Animal Care & Use Committee (IACUC) of Duke University & Duke University Medical Center.

Media used to screen the cardio-inducing effect of the TF modules included: basic fibroblast growth medium (10%FBS, DMEM.HG, Glutamax, NEAA, sodium pyruvate, βMercaptoethanol, gentamicin), cardiomyocyte growth medium (Lonza, CC-4515), and low serum growth medium (Lonza, CC-3186). Media also contained doxycycline for induction of TF expression (10 µg/ml, Sigma, D9891), and valproic acid (0.5 mM, Sigma, P4543). We also tested the cardio-inducing effect of JAK inhibitor (0.5 µM, EMD4Biosciences, 420099-500 UG). Extracellular matrix proteins screened included porcine skin gelatin (Sigma, G1890), and poly-L-lysine (Sigma, P4707).

Primary cultures of MEFs isolated from the transgenic mice were epigenetically reprogrammed into induced pluripotent stem cells using a previously described polycistronic inducible-expression lentiviral vector [18] (FU.tet.on.OSKM). Briefly, following cell transduction with the lentiviral vector, cells were cultured in a reprogramming medium (20%FBS, DMEM.HG, NEAA, Sodium Pyruvate, Glutamax, βMercaptoethanol, Gentamicin, valproic acid, and leukemia inhibitory factor) in the presence of doxycycline. Doxycycline was removed 7 days post induction and individual colonies of induced pluripotent stem cells were mechanically picked and expanded. The cells were subsequently differentiated into cardiomyocytes using the previously described [19] hanging droplet technique for embryoid body formation.

### DNA Vectors, Viral Particle Production, and Cell Transductions

DNA vectors designed for the production of lentiviral particles that allow the inducible expression of cardiac TFs in the targeted transduced cell type were assembled using a previously described lentiviral vector [20] which controlled the expression of *OCT4* (Addgene plasmid 19778, FU.tet.on.OCT4). Fully sequenced TF cDNA clones were purchased from Open Biosystems and cloned in the FU.tet.on plasmid: GATA4 (BC105108, 5′ & 3′ EcoRI), HAND1 (BC021190, 5′ EcoRI & 3′ AcuI), HAND2 (BC101406, 5′ & 3′ EcoRI), MEF2C (BC026341, 5′ AcuI & 3′ SpeI), Mesp1 (BC125505, 5′ & 3′ EcoRI), MYOCD (BC126307, 5′ SpeI & 3′ NotI), NKX2-5 (BC025711, 5′ EcoRI & 3′ NotI), SMARCD3 (Addgene Plasmid 21036, 5′ & 3′ EcoRI), SRF (BC048211, 5′ EcoRI & 3′ XhoI), and TBX5 (BC027942, 5′ & 3′ EcoRI). For the purposes of this project we chose to purchase only fully sequenced and validated cDNA clones from the Open Biosystems Mammalian Gene Collection. The human homolog of Mesp1 was not available when we were initiating this study and thus we purchased the mouse homolog and performed all our experiments with mouse Mesp1. To ensure the appropriateness of utilizing the human gene homologs we compared their protein sequence to that of their mouse counterparts using a multiple sequence alignment tool (http://www.ebi.ac.uk/Tools/msa/clustalw2/). Through the ClustalW2 protein sequence analysis we determined that there is a 100% homology in the functional protein domains (i.e. DNA binding domains) of both the mouse and human protein homologs. We used a previously described lentiviral inducible expression vector in order to reprogram MEFs into induced pluripotent stem cells: FU.tet.on.OSKM [18] (Addgene plasmid 20321).

The lentiviral reporter vectors were constructed or acquired as follows: the previously described mouse Nkx2-5.Hsp68.eGFP reporter plasmid [19] was cloned into the FUGW [21] lentiviral vector (Addgene plasmid 14883) replacing the ubiquitin promoter and GFP. The previously described 2.7 Kb rat Myl2 promoter element [22] and mCherry fluorescent protein gene were cloned in the pRRLSIN.cPPT.PGK-GFP.WPRE lentiviral vector (Addgene plasmid 12252) replacing PGK-GFP. The human TNNT2.copGFP lentiviral reporter vector was acquired from System Biosciences (Catalog # SR10012). The mouse Myh6.eGFP lentiviral reporter vector was previously described [23] (Addgene plasmid 21229). To construct the FUW.GCaMP3 lentiviral vector (constitutive expression of GCaMP3) we replaced GFP in FUGW [21] (Addgene plasmid 14883) with GCaMP3 [24] (Addgene vector 22692). To construct the TNNT2.GCaMP3 reporter vector, the ubiquitin promoter-rtTA cassette of FUdeltaGW-rtTA (Addgene plasmid 19780) was excised and replaced with the human TNNT2 promoter and a Gateway cassette (PCR-amplified from pEF-DEST51, Invitrogen) to create TNNT2-Gateway. The GCaMP3 cassette was cloned into pDONR221 (Invitrogen), and subsequently into TNNT2-Gateway, via Gateway recombination. To construct the FU.tet.on.GFP reporter vector we cloned the GFP fluorescent protein gene in the FU.tet.on inducible expression lentiviral vector. We acquired the FUW.M2rtTA lentiviral vector [25] from Addgene (Addgene plasmid 20342).

To produce viral particles we used a previously described second generation lentivirus production system utilizing the psPAX2 (packaging vector, Addgene plasmid 12660) and pMD2.G (envelope vector, Addgene plasmid 12559) vectors (Dr. Didier Trono). We followed a procedure similar to the one described by Maherali N et al [20]. Briefly HEK293T cells were maintained in a standard growth medium and expanded in T-75 tissue culture flasks pre-coated with a 0.1% solution of porcine gelatin (Sigma). Cells were allowed to reach 90% confluence at which point they were transfected in the presence of Opti-MEM® (Life Technologies) with a total of 24 µg of the three lentiviral vectors (12 µg expression vector, 7.7 µg of psPAX2 and 4.3 µg of pMD2.G) using Lipofectamine 2000 (Life Technologies). The supernatant containing the viral particles was collected at 48 and 96 hours following initial transfection with a final volume of 20 ml. The supernatant was subsequently concentrated to a final volume of approximately 300 µl using Amicon Ultra-15 centrifugal filter unites (Millipore) and stored at 4°C for immediate use or in small aliquots at −80°C for long term use.

To transduce primary MEFs, the cells were plated at a density of approximately 10,000 cells/cm^2^ is 6-well plates. The next day 1 ml of transduction medium containing 10 µl of each viral concentrate and 8 µg/ml sequabrene (Sigma) was used to transduce the cells. The same method was used to transduce the cell line NIH3T3 although we plated the cells at a concentration of 2,000 cells/cm^2^ is the 6-well plates as they have a much higher proliferation rate. To ensure homogeneous transduction cells were first transduced with FUW.M2rtTA and the various reporter vectors, and subsequently passaged into new 6-well plates in order to be transduced with the various combinations of transcriptional modules. Transgene overexpression was induced by the addition of Doxycycline in the culture medium. Qualitative RT-PCR analysis was performed to ensure transcriptional expression of the delivered transgenes (Figure S1).

### Imaging, Fluorescence-activated Cell Sorting, and Immunofluorescence

Fluorescent cell imaging was performed on either a Nikon Eclipse TE2000-U using a Roper Scientific CoolSnap HQ camera and the NIS Elements software suite or a Zeiss 510 inverted confocal microscope. FACS sorting was performed on either a DiVa sorter (Becton Dickinson) or a FACStar (BD Biosciences). FACS analysis was performed on a FACSCanto flow cytometer (BD Biosciences). For all the FACS experiments using the non-transgenic mouse embryonic fibroblasts, we used two cell populations as the negative control: negative control group 1 was comprised of fibroblasts transduced with only the FUW.M2rtTA and no reporter vector, and negative control group 2 was comprised of fibroblasts transduced with both the FUW.M2rtTA and one of the four reporter vectors. The gates which we defined for each of the reporter vectors were established as to not include any cells within negative control group 1. Meta-analysis of acquired FACS data was performed using Cyflogic (www.cyflogic.com) and Microsoft Excel. Primary antibodies used for immunofluorescence included: anti-Tnnt2 (R&D Systems, MAB1874), anti-Actn2 (Sigma, A7811), anti-Myh6 (Abcam, ab15), anti-Myl2 (Abcam, ab48003), anti-Acta2 (Abcam, ab5694), anti-Nppa (Abcam, ab14348), anti-Gja1 (Santa Cruz, SC-9059). Secondary antibodies used were raised in either chicken or goat against mouse or rabbit antibodies. They were purchased from Life Technologies and conjugated to Alexa Fluor® 568 (red), Alexa Fluor® 488 (green), or Alexa Fluor® 350 (blue). Cell nuclei were detected using DAPI (Life Technologies, D1306). The effect of valproic acid addition on the level of cardio-induction was determined by counting Tnnt2 or Actn2 positive cells in the presence or absence of valproic acid.

### Relative Gene Expression Analysis

Primers were designed using NCBI primer-BLAST. In order to avoid polymerization of non-specific DNA amplimers, when applicable primers were required to span an exon-exon junction and the primer pair was required to be separated by at least one intron on the corresponding genomic DNA (Table S1).

To perform qualitative RT.PCR analysis and ensure low level of DNA amplification without signal saturation, following total RNA isolation (Qiagen, 74104) a one-step RT.PCR kit (Qiagen, 21212) was utilized with 23 cycles of amplification for Gapdh and 26–28 cycles (BioRad, MyCycler) for the rest of the genes analyzed. Quantitative RT.PCR analysis was performed on either an ABI 7300 or 7900HT real time thermocycler using the QuantiTect SYBR Green one-step RT.PCR kit (Qiagen, 204243). While running the polymerase reactions on the real time thermocyclers we included the option of getting a dissociation curve and also run the final reaction product on agarose gels in order to ensure that the only amplimers detected and measured were the expected ones. The SDS software (ABI, version 1.4 or 2.4) was used to analyze the raw data and then additional analysis was performed on Microsoft Excel. Relative quantification was performed using the ΔΔCt method and statistical significance was determined using the T-Test.

TaqMan Low Density Array Cards (TLDA) were custom designed using TaqMan probes (Table S1). Reverse transcription was performed using the high capacity RNA-to-cDNA kit (ABI, 4387496), and real time PCR was performed using the TaqMan Gene Expression Master Mix (ABI, 4369016) on an ABI 7900HT thermocycler (Duke genomics facility). Data analysis was performed using ABI RQ Manager 1.2.1, ABI SDS (2.4), and Microsoft Excel. Relative quantification was performed using the ΔΔCt method and statistical significance was determined using the T-Test.

### Electrophysiological Characterization

Sharp microelectrodes were fabricated from standard wall borosilicate glass capillary tubes (Sutter BF 100-50-10, Sutter Instruments) using a P-97 Sutter micropipette puller to generate electrodes with tip resistances between 50 and 70 MΩ when backfilled with 3 M KCl. The electrodes were connected to the headstage of a Multiclamp 700B amplifier (Axon Instruments, Inc.) using a silver chloride wire embedded within the micropipette holder. A reference silver chloride wire was connected to the bath chamber through an agar bridge. Cell cultures were perfused with warm (35–37°C) Tyrode’s solution consisting of (mM) 135 NaC1, 5.4 KCl, 1.8 CaCl_2_, 1.0 MgCl_2_, 0.33 NaH_2_PO_4_, 5 HEPES, and 5 glucose; pH was adjusted to 7.4 with NaOH.

Membrane potential measurements were made using the current clamp mode of the Multiclamp 700B amplifier after electrode potential offset and capacitance were neutralized. Resting membrane potential was recorded from GFP(+) and GFP(**−**) cells (TNNT2.copGFP or TNNT2.GCaMP3) after the establishment of stable intracellular impalement. Data were sampled at 20 kHz and low-pass filtered using a 4.5 kHz Butterworth filter. Recordings were digitized using a BNC-2090 adaptor and NIDAQ-MX computer interface (National Instruments Corporation, Austin, TX). Signals were recorded and analyzed using WinWCP software (John Dempster, University of Strathclyde).

Videos of cells exhibiting GCaMP3 flashing were captured using a Nikon Eclipse TE2000-U with a Roper Scientific CoolSnap HQ camera and the NIS Elements software suite. The video files were subsequently analyzed with FIJI [26] as a stack of images to determine the average signal intensity in a region of interest within each cell. The average intensity for a given region of interest was normalized to the lowest value acquired and then plotted against the acquisition time of each frame. Spectral analysis was performed on linearly resampled (1 Hz) time series using Welch’s method [27]. The 256-point fast Fourier transform was repeatedly computed with 50% overlap between adjacent segments. Then the spectral power of each segment was computed and averaged. Hanning window filtering was applied to avoid spectral leakage.

### Microarray Gene Expression Analysis

MEFs were first transduced with the M2rtTA lentivirus and subsequently divided into four groups which were transduced with TF group 1 (*G_4_T_5_M_C_*), TF group 2 (*G_4_T_5_M_C_M_1_S_3_*), TF group 3 (*G_4_T_5_M_C_M_D_S_F_M_1_S_3_*), or just FUW.M2rtTA (negative control). Induction of transgenic TF expression was initiated two days following lentiviral transduction at which point the standard MEF medium was supplemented with an additional 10%FBS, VPA (0.5 mM), and Dox (10 µg/ml). Total RNA was isolated 7 days following induction of TF expression (Qiagen, RNeasy) and expression of the TF was confirmed using qualitative RTPCR. 200 ng of RNA was prepared for microarray hybridization (Life Technologies, MessageAmp Premier RNA amplification kit). For the purposes of this experiment we utilized twelve (12×) Affymetrix Mouse Genome 430A 2.0 microarrays (each cell group was repeated in triplicate) and the entire process of RNA amplification, hybridization, and raw data preparation was performed by the Duke University microarray core facility. The raw data files and experimental description have been submitted to NCBI Gene Expression Omnibus (GSE44401). For the heart and MEF control samples we acquired the CEL raw data files from NCBI Gene Expression Omnibus. Heart control (430 2.0 chip): GSM206354, GSM206355, GSM206356, GSM252113, GSM252114, GSM252115, GSM275315, GSM275316, GSM275317, GSM311517, GSM311518, GSM311519, GSM311520, GSM311521, GSM311522, GSM311523, GSM311524, GSM311525, GSM311526, GSM311527, GSM466341, GSM466348, GSM466349, GSM496724, GSM496725, GSM496726, GSM756426, GSM756427, GSM756428. MEF control (430 2.0 chip): GSM198070, GSM198072, GSM276426, GSM276427, GSM276428, GSM409451, GSM409454.

The main part of data analysis was performed using the Partek Genomics Suite. First, the raw data (CEL files) were imported and normalized using the RMA algorithm and subsequently ANOVA statistical analysis was performed on the entire data set searching for significant differences between any one of the three TF groups and the negative control. Subsequently significantly upregulated or downregulated genes were identified based on the fact that p-value<0.05 and Fold Change< or >1.5. Hierarchical clustering analysis and self-organizing map analysis were performed within the software suite. For the purpose of integrating the data originating through Partek into genetic pathways, we performed pathway analysis by utilizing the Thomson Reuters Metacore GeneGo pathway suite. The Excel add-in statistical analysis suite XLSTAT was used to perform the principal component analysis.

## Results

### Screening Assay for Determining the Cardio-Inducing potential of TFs

Ten TFs, previously shown to play a role during developmental cardiogenesis (*Ms Mesp1, Hs SMARCD3, Hs MYOCD, Hs SRF, Hs NKX2-5, Hs HAND1, Hs HAND2, Hs GATA4, Hs TBX5, Hs MEF2C*), were screened for their cardio-inducing capacity. To achieve efficient cellular transduction we used an inducible lentiviral-based gene delivery system (tet-on). Activation of transgene overexpression was depended on doxycycline addition. The initial screen was performed with two output readouts: 1) cardiac-specific reporter-based assay (lentivirally-delivered reporter, Myh6.eGFP [23]), and 2) quantitative transcriptional activation of cardiomyocyte-specific gene loci (*Myh6*, *Myl2*, *Tnnt2*). The ten TFs were grouped and delivered within four TF modules based on previous evidence demonstrating their role during developmental cardiogenesis: *M_1_S_3_* [28]–[30], *M_D_S_F_* [31]–[33], *G_4_T_5_M_C_* [34], [35], and *N_5_H_1_H_2_* [34], [35].

**Figure 1:**
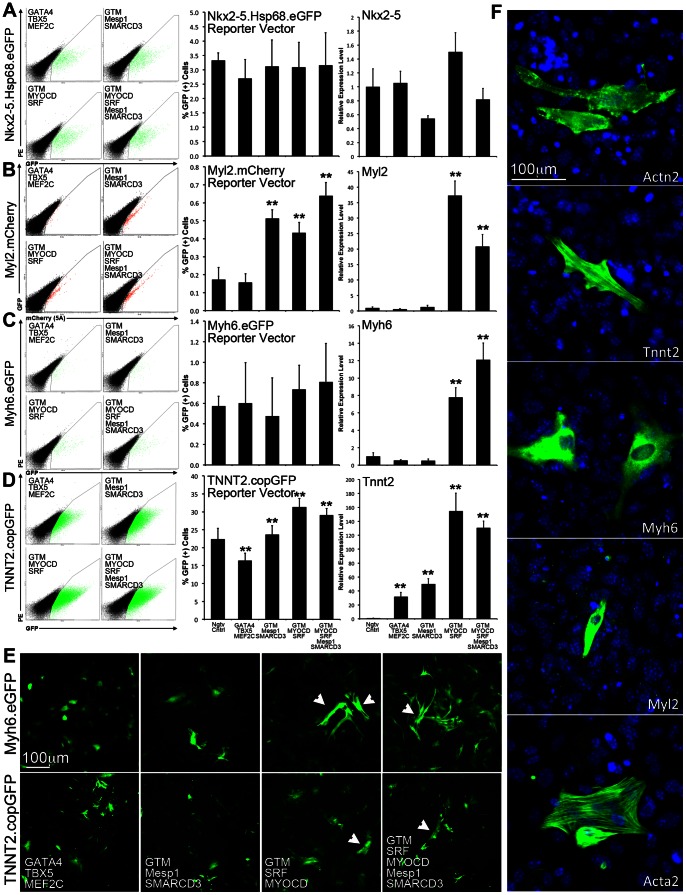
Determination of transcriptional cardio-inducing effect through detection of reporter vector activity and relative quantification of endogenous gene expression levels. **A–D.** Prior to transduction and induction of TF module expression, MEFs were transduced with four different reporter vectors (Nkx2-5.Hsp68.eGFP, Myl2.mCherry, Myh6.eGFP, TNNT2.copGFP). MEFs were subsequently transduced with *G_4_T_5_M_C_*, *G_4_T_5_M_C_M_1_S_3_*, *G_4_T_5_M_C_M_D_S_F_*, or *G_4_T_5_M_C_M_D_S_F_M_1_S_3_*. Negative control MEFs were only transduced with reporter vector and the M2rtTA-expressing lentivirus. The gates were defined using MEFs that were only transduced with the M2rtTA expressing lentivirus and no reporter vector. Following 7 days of induction of TF module expression, the fraction of cells expressing either GFP or mCherry was determined by FACS analysis. The relative gene expression level of the gene used in each of the reporter vectors was determined using quantitative RT.PCR analysis (endogenous locus). Results for both FACS and RTPCR are based on biological triplicates. Error bars represent calculated standard deviation (One *for p-value <0.05, Two *for p-value <0.01). **E**. Live imaging of cells transduced with either Myh6.eGFP, or TNNT2.copGFP. White arrows point to examples of brightest and elongated cells detected in MEFs transduced with either *G_4_T_5_M_C_M_D_S_F_*, or *G_4_T_5_M_C_M_D_S_F_M_1_S_3_*. **F**. Detection of cardiac protein expression (Actn2, Tnnt2, Myh6, Myl2, Acta2) in MEFs transduced with *G_4_T_5_M_C_M_D_S_F_M_1_S_3_* using immunofluorescence.

Based on the initial screening results demonstrating a consistent cardio-inducing effect (Figure S2), three TF modules were selected for further experiments to determine their cardio-inducing potential: *G_4_T_5_M_C_*, *M_1_S_3_*, *M_D_S_F_*. We first examined whether we could detect and quantify a cardio-inducing effect using lentivirally-delivered reporter vectors (Figure 1A–D, Nkx2-5.Hsp68.eGFP [19], Myl2.mCherry [22], Myh6.eGFP [23], TNNT2.copGFP). To validate the acquired output from each reporter vector we measured the level of transcriptional activation of the correspondent endogenous locus (*Nkx2-5*, *Myl2*, *Myh6*, *Tnnt2*).

We determined that when using the Nkx2-5 reporter, TF overexpression did not induce an increase in GFP(+) cells, while detected a significant GFP(+) cell fraction (3.32±0.26%) in the negative control, indicative of the reporter vector’s leakiness. No significant change was detected when measuring the gene expression levels of endogenous *Nkx2-5*. Data acquired using the Myl2 reporter closely resembled the levels of endogenous gene expression, although we detected a significant discrepancy when using a particular TF combination (*G_4_T_5_M_C_M_1_S_3_*). This may be indicative of direct binding and activation of *Myl2* transcription, by *M_1_* or *S_3_*. Background Myl2 reporter activity was lower as compared to that of the Nkx2-5 reporter (0.28±0.07%). With the Myh6.eGFP reporter we detected a non-significant increase in the GFP(+) cell fraction for two TF combinations. The same two combinations produced a significant increase in endogenous *Myh6* expression. Finally, the output measured when using the TNNT2.copGFP reporter resembled the endogenous Tnnt2 expression level for the two combinations containing MYOCD and SRF although we detected a significant GFP(+) cell fraction (22.38±3.04%) in the negative control. A significant upregulation in *Tnnt2* expression was detected with all four TF module combinations, although for two of them (same as for *Myh6*) the cardio-inducing effect was stronger (*G_4_T_5_M_C_M_D_S_F_*, and *G_4_T_5_M_C_M_D_S_F_M_1_S_3_*).

In addition to reporter vector activity, we also looked for morphological changes in the transduced cell populations (Figure 1E). By day 7 we readily detected brighter and elongated GFP(+) cells clearly distinguishable from the rest of the GFP(+) cells. This observation was particularly evident in MEFs transduced with either *G_4_T_5_M_C_M_D_S_F_*, or *G_4_T_5_M_C_M_D_S_F_M_1_S_3_*.

To examine whether cardiac specific proteins were expressed in the transduced MEFs we performed immunofluorescence using antibodies against proteins Actn2, Tnnt2, Myh6, Myl2, and Acta2 (Positive control: Figure S3). At day 14 we detected a few positive cells in the group transduced with all the TF module combinations (Figure 1F). The expressed proteins did not organize in a cross-striated cytoskeletal pattern usually detected in functional cardiomyocytes.

We also tested whether valproic acid, previously shown to increase the efficiency of iPS cell derivation [36], could affect the TF cardio-inducing effect. Simultaneous TF overexpression and valproic acid treatment significantly improved the overall cardio-inducing effect by approximately two-fold as determined by the fraction of cells expressing either Actn2 or Tnnt2 (2.07±0.51 fold, p-value: 0.004) (Figure S4).

In addition to the morphological changes detected in a small cell fraction (elongation, and increased levels of GFP expression), we also observed a decrease in cell proliferation as compared to the negative control. Moreover, the TF-expressing cells and especially the ones transduced with the *M_D_S_F_* TF module, did not dissociate adequately with trypsin/EDTA and remained aggregated. Even when using a customized enzymatic solution, the dissociated cells did not attach once replated, and eventually underwent apoptosis. This was especially evident within the brightest GFP(+) cell fraction. We hypothesized that dissociation and expansion difficulties, coupled with the leaky activity of the reporter vectors were applying a negative selection on the cells undergoing reprogramming. To examine this we performed relative gene expression analysis on sorted and expanded GFP(**−**) and GFP(+) cells (Myh6.eGFP, Figure S5). We did not detect a significant upregulation of cardiac genes in the GFP(+) cell population further validating our hypothesis.

### Expression of Mesp1, SMARCD3, MYOCD, and SRF Significantly Enhance the Cardio-inducing Effect of GATA4, TBX5, and MEF2C

To further elucidate the cardio-inducing effect of the three TF modules while avoiding interference from the reporter vectors, we used MEFs from transgenic mice expressing GFP and puromycin *N*-acetyl-transferase (PAC) under the control of the cardiac mouse myosin heavy chain promoter [37]. First, we confirmed the lack of GFP expression in cultures of isolated MEFs, and tested whether the cells retained the capacity to express GFP in a cardiac-specific manner when epigenetically reprogrammed. Following iPS cell derivation and differentiation [18] [19], we confirmed specific GFP expression in spontaneously contracting cardiomyocytes (Figure S6, Movie S1).

**Figure 2:**
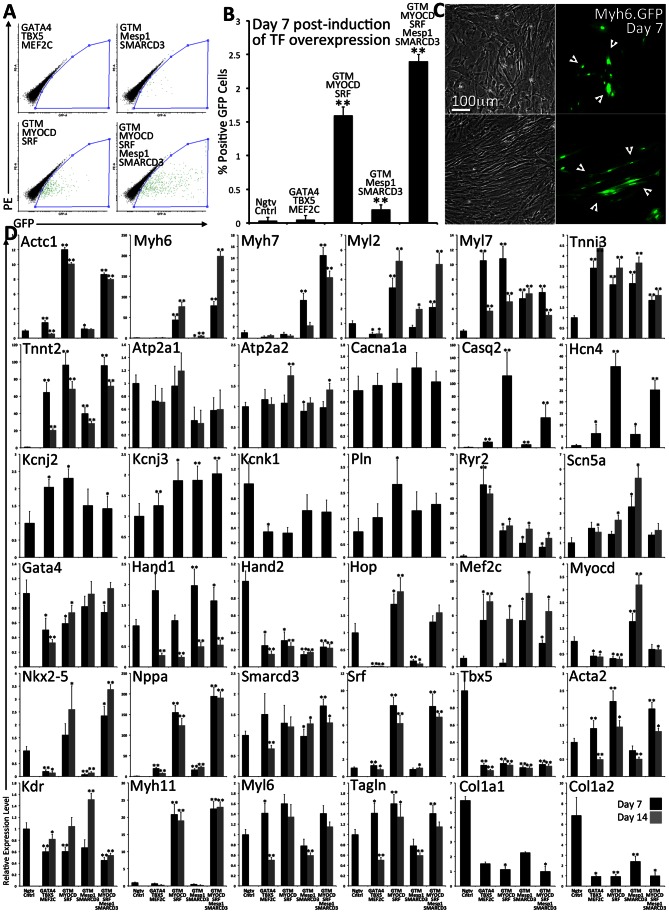
Determination of the cardio-inducing effect of four combinations of TF modules in primary transgenic-mouse MEFs. **A–B**. Primary MEFs were isolated and expanded from transgenic mice where the expression of GFP is controlled by the myosin heavy chain promoter element. MEFs were transduced with FUW.M2rtTA and one of four transcriptional module combinations: *G_4_T_5_M_C_*, *G_4_T_5_M_C_M_1_S_3_*, *G_4_T_5_M_C_M_D_S_F_*, *G_4_T_5_M_C_M_D_S_F_M_1_S_3_*. Following 7 days of induction of TF overexpression the fraction of cells expressing GFP was determined using FACS. Results are based on biological triplicates. Error bars represent calculated standard deviation (One *for p-value <0.05, Two *for p-value <0.01). **C**. GFP(+) MEFs were readily detected within 7 days of TF overexpression. We detected GFP(+) cells that were smaller in size (upper panels) or larger and elongated (lower panels). **D**. RNA was isolated at either 7 (black) or 14 days (gray) post induction of TF overexpression. Using quantitative RT.PCR and custom-designed TaqMan Low Density Array plates we measured the relative gene expression levels normalizing to negative control MEFs (FUW.M2rtTA only). Error bars represent calculated standard deviation (One *for p-value <0.05, Two *for p-value <0.01).

GFP expression was detected as early as 2 days post induction of TF overexpression in MEFs transduced with either *G_4_T_5_M_C_M_D_S_F_* or *G_4_T_5_M_C_M_D_S_F_M_1_S_3_*. By day 7 GFP expression was determined to be 1.60±0.12% and 2.40±0.11% respectively for the two TF groups (Figure 2A, B). This level of GFP expression was highly significant as compared to the negative control (0.03±0.05%). Only rare events of GFP(+) cells were detected in MEFs transduced with only *G_4_T_5_M_C_* (0.05±0.06%) and a slightly higher fraction in MEFs transduced with *G_4_T_5_M_C_M_1_S_3_* (0.20±0.07%), which was not significant. It is noteworthy that we detected two main cell morphologies: small and round (Figure 2C, lower) or large and elongated cells (Figure 2C upper) which could be attributed to an effect caused by TF overexpression, or an influence from the surrounding cells.

We next examined whether GFP expression correlated with changes at the gene expression level of cardiac related genes (Figure 2D, relative mRNA quantification). We custom-designed TaqMan Low Density Array plates allowing us to screen the transcriptional output of a large number of genes. Some of the overexpressed TF transgenes are usually active both during early cardiac development as well as in mature cardiomyocytes. The array plates were designed with the aim to detect both markers expressed in immature and mature cardiomyocytes. It is important to note that this analysis was performed on RNA isolated from the total cell population of each of the four TF groups and not from cells sorted based on GFP expression. This was done mainly because two of the TF groups (*G_4_T_5_M_C_* or *G_4_T_5_M_C_M_1_S_3_*) did not induce any significant GFP expression in the transduced MEFs.

In general, using temporal gene expression analysis (7 or 14 days of TF overexpression), we recorded a significant effect in the transcriptional output (upregulation and downregulation) for some genes whereas for others no significant change was detected. This effect also varied amongst the different TF groups overexpressed. More specifically we detected a significant upregulation in the expression level of cardiomyocyte specific cytoskeletal proteins *Actc1*, *Myh6*, *Myh7*, *Myl2*, *Tnni3*, and *Tnnt2*. This effect was particularly evident in MEFs transduced with either *G_4_T_5_M_C_M_D_S_F_* or *G_4_T_5_M_C_M_D_S_F_M_1_S_3_*. The recorded differences also remained constant over time for most of the genes examined. When measuring the gene expression level of endogenous cardiac transcription factors we did not detect any large changes as compared to the control sample. It is noteworthy to mention that endogenous *Mef2c* expression was significantly increased, whereas *Gata4* expression was slightly decreased, and Tbx5 expression was largely decreased for all TF groups used­. We also detected a significant decrease for *Hand1* (Day 14) and *Hand2* (Days 7 & 14), and a small, but significant increase for *Nkx2-5* and *Srf* expression for two of the TF groups. *Myocd* was upregulated only in cells transduced with *G_4_T_5_M_C_M_1_S_3_*. *Hop* expression was significantly decreased in cells transduced with either *G_4_T_5_M_C_M_D_S_F_* or *G_4_T_5_M_C_M_D_S_F_M_1_S_3_*. The same analysis was also performed using wild-type MEFs transduced with three of the TF module groups and the results closely correlate with the ones presented in Figure 2 (Figure S7).

We also examined the expression levels of genes that play a role in the electrophysiological function of cardiac myocytes. We did not detect any significant variations in the relative gene expression level for *Atp2a1*, *Atp2a2*, *Cacna1a*, *Kcnj2*, *Kcnj3*, *Kcnk1*, and *Pln*. Genes with significant expression upregulation included *Casq2*, *Hcn4*, and *Nppa* particularly in cells transduced with either *G_4_T_5_M_C_M_D_S_F_* or *G_4_T_5_M_C_M_D_S_F_M_1_S_3_*. The TF module *G_4_T_5_M_C_* had the strongest effect in inducing upregulation of *Ryr2*. We also detected a low level of *Scn5a* upregulation amongst all the TF groups, with *G_4_T_5_M_C_M_1_S_3_* being the most potent.

The TFs used are also highly expressed during cardiac development in cardiac progenitors [19], [38], which have the capacity to differentiate into cardiomyocytes, smooth muscle and endothelial cells. When examining the expression levels of endothelial or smooth muscle specific genes, we detected small or no changes in the expression of *Acta2*, *Kdr*, *Myl6*, and *Tagln*. We detected a significant increase in expression of smooth muscle *Myh11* in cells transduced with *G_4_T_5_M_C_M_D_S_F_* or *G_4_T_5_M_C_M_D_S_F_M_1_S_3_*. Finally, we detected a significant decrease in the expression level of fibroblast-specific genes *Col1a1* and *Col1a2* [17].

We also set out to determine the relative gene expression levels for the same genes described above when comparing positive control hearts to MEFs. Relative fold change for these genes was calculated using published Affymetrix microarray gene expression data (Table S2). The largest and most significant fold change was determined for genes *Myh6*, *Myl2*, *Tnni3*, *Pln*, *Nppa*, *Ryr2*, *Tnnt2*, *Actc1*, *Casq2*, *Atp1a2*, and *Myl7*. Significantly downregulated genes included *Acta2*, *Tagln*, *Col1a2*, and *Col1a1*. No significant change in gene expression was determined for *Kcnj2*, *Kcnk1*, *Hand1*, *Hand2*, *Srf*, *Myl6*, and *Hcn4*. Although the fold change determined when comparing cardiac tissue versus MEFs is significantly higher than the one determined when comparing the transduced MEFs versus the negative control it is noteworthy to mention that the RNA used in our experiments was isolated from the total population of transduced cells and not an enriched cell population.

**Figure 3:**
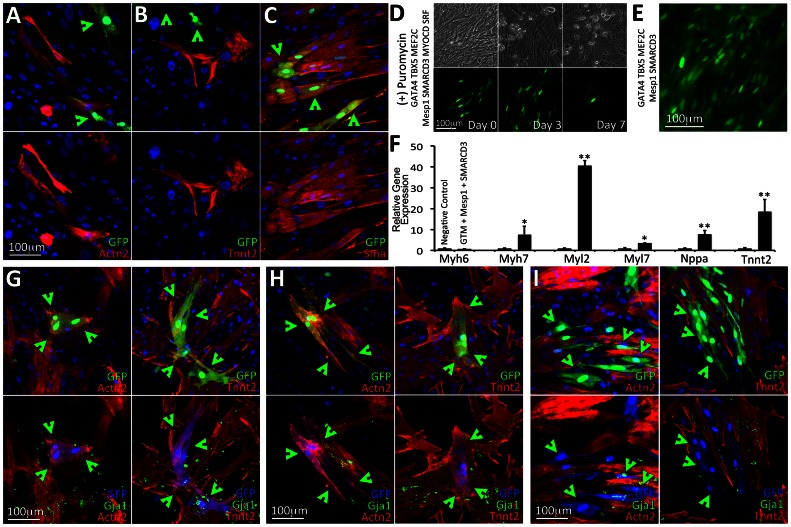
Assaying the level of cardiac protein expression in reprogrammed MEFs and following genetic selection **A–C**. GFP(+) cells were readily detected in MEFs transduced with either *G_4_T_5_M_C_M_D_S_F_*, or *G_4_T_5_M_C_M_D_S_F_M_1_S_3_* within 2 days of induction of TF expression. Only rare GFP(+) cells were detected in MEFs transduced with either *G_4_T_5_M_C_* or *G_4_T_5_M_C_M_1_S_3_*. Using immunofluorescence analysis (day 7) we detected cells staining positive for cardiac antigens Actn2 or Tnnt2 although GFP expression was not always co-localized with these two proteins. We also detected co-localization of GFP expression with Acta2. The GTM label refers to the TF module containing GATA4, TBX5, and MEF2C. **D–F**. Puromycin was used to positively select for cells activating the transgenic cardiac promoter element in isolated MEFs following transduction and induction of expression of the four TF module combinations. We observed significant death of non-GFP expressing cells following 3 days of low-level puromycin selection (induction day 7+3). In MEFs transduced with either *G_4_T_5_M_C_M_D_S_F_* or *G_4_T_5_M_C_M_D_S_F_M_1_S_3_* GFP(+) cells remained alive but did not proliferate. Following 7 days of puromycin selection, GFP(+) cells for those two TF module combinations peeled off the plastic surface and subsequently underwent apoptosis (D). Rare GFP(+) cells were detected in MEFs transduced with *G_4_T_5_M_C_M_1_S_3_*. The GFP(+) cells proliferated in the presence of puromycin (E). Relative gene expression analysis performed on puromycin-selected surviving GFP(+) cells in MEFs transduced with *G_4_T_5_M_C_M_1_S_3_* (F). (**G–I**). Cocultures of transgenic MEFs transduced with the four combinations of TF modules established with freshly isolated neonatal rat ventricular myocytes (NRVMs). Following induction of TF expression for 7 days we assayed cardiac protein expression (Actn2, Tnnt2) and gap junction formation (Gja1). In cocultures where MEFs were transduced with either *G_4_T_5_M_C_M_D_S_F_* or *G_4_T_5_M_C_M_D_S_F_M_1_S_3_* we detected three distinct staining patterns. Firstly, we detected GFP(+) cells with Actn2 or Tnnt2 co-localization. Importantly the cardiac proteins in these cells formed cross-striations (G). Secondly, we detected GFP(+) cells with Actn2 or Tnnt2 co-localization, where however the two cardiac proteins remained unorganized and no cross-striations where detected (H). Thirdly, we detected GFP(+) cells which were negative for either Actn2 or Tnnt2 expression (I). Gja1 staining did not indicate noticeable gap junction formation between the GFP-expressing cells and NRVMs.

We used antibodies against Actn2, Tnnt2, or Acta2 to determine whether the MEFs isolated from transgenic animals were expressing and organizing these proteins in cross-striated sarcomeres (Figure 3A–C). We readily detected cells staining positive for all three of these proteins, however the staining pattern was not cross-striated indicating again an immature expression of these proteins. Actn2 and Tnnt2 expression did not always coincide with GFP expression, indicating, that some cells expressed these proteins without activating the Myh6.eGFP reporter locus.

We tested whether we could select the GFP(+) MEFs based on the fact that the same cardiac promoter element also controlled expression of PAC (Figure 3D). We added a low concentration of puromycin in the culture medium and monitored the cells temporally. Almost immediately (Day 1–2) it became obvious that puromycin was toxic for the majority of GFP(**−**) cells, while the GFP(+) cells remained unaffected. We did not observe any proliferation in the GFP(+) cell population. On the contrary as more of the surrounding GFP(**−**) cells died, it led to the detachment of GFP(+) cells. This may be an indication that MEFs undergoing reprogramming require a supporting niche provided by the surrounding fibroblasts to survive. We also detected proliferation in GFP(+) MEFs transduced with either *G_4_T_5_M_C_M_1_S_3_* or only *M_1_S_3_*, suggesting that the combination of Mesp1 and SMARCD3 may be inducing transcriptional activation of the transgenic promoter element while at the same time inducing proliferation (Figure 3E). Gene expression analysis performed on proliferative GFP(+) cells demonstrated a large and significant upregulation of endogenous *Myl2*.

Since the puromycin selection experiments suggested that the niche in which MEFs were being cultured may play an important role for their survival and possibly successful cellular reprogramming we tested whether establishing cocultures of pre-transduced MEFs with NRVMs would have an additional cardio-inducing effect. GFP(+) MEFs were readily detected within 2 days of induction of TF expression in cocultures when transduced with either *G_4_T_5_M_C_M_D_S_F_* or *G_4_T_5_M_C_M_D_S_F_M_1_S_3_*. At day 7 we detected three unique phenotypes for the GFP(+) cells: 1) the GFP was co-localized with either Actn2 or Tnnt2 and the two cytoskeletal proteins organized in a cross-striated pattern (Figure 3G); 2) the GFP co-localized with either Actn2 or Tnnt2 but the two proteins were not organized in a cross-striated manner (Figure 3H); and 3) the GFP did not co-localize with either of the two cardiac proteins (Figure 3I). Connexin 43 (Gja1) that should be present along the borders of cardiomyocytes was not detected between the border of the transduced MEFs and NRVMs. We did not assay for fusion events between the GFP(+) cells and the NRVMs.

### Global Transcriptome and Gene Pathway Analysis

**Figure 4:**
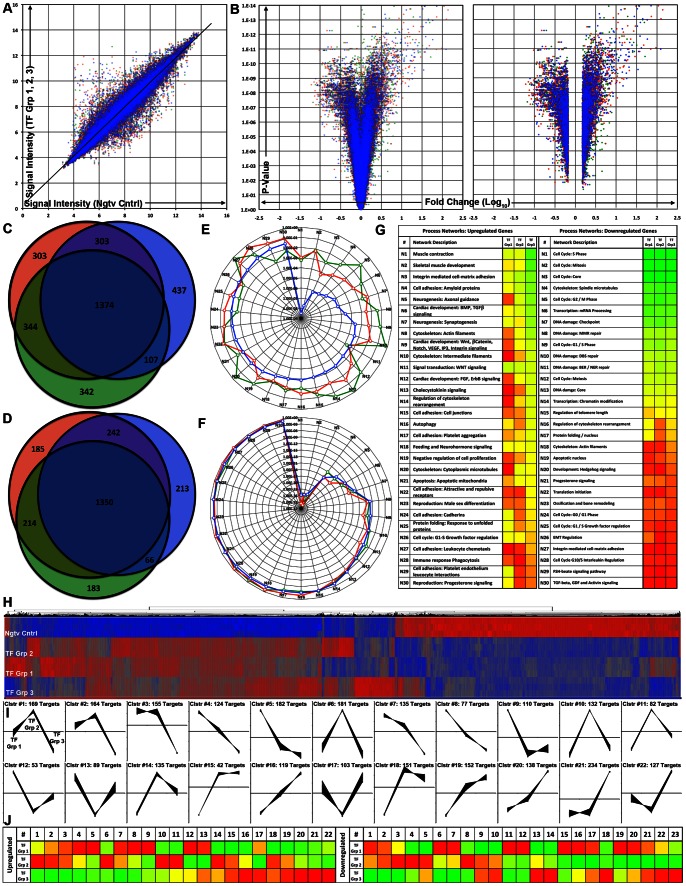
Microarray gene expression analysis performed on populations of reprogrammed MEFs **A**. Plot of signal intensity ratios for each individual chip probe when comparing MEFs transduced with any of the three combinations of TF modules to the negative control (TF Group 1 *G_4_T_5_M_C_* (Green), TF Group 2 *G_4_T_5_M_C_M_1_S_3_* (Red), TF Group 3 *G_4_T_5_M_C_M_D_S_F_M_1_S_3_* (Blue)). **B**. Volcano plots displaying the relationship between the calculated fold change for each individual chip probe (when comparing each of the treated cell groups and the negative control) versus the P-value determined using ANOVA statistical analysis. The graph on the left contains all the chip probes whereas the graph on the right contains only probes that are significantly upregulated or downregulated (Fold Change<or >1.5, p-value <0.05) (TF Group 1 (Green), TF Group 2 (Red), TF Group 3 (Blue)). **C–D**. Venn diagrams displaying the numbers of common probe IDs for either significantly upregulated (C) or significantly downregulated chip probes (D) when comparing each of the treated cell groups and the negative control. The 1374 chip probes common for significantly upregulated genes correspond to 1065 genes whereas the 1350 chip probes common for the significantly downregulated genes correspond to 980 genes (TF Group 1 (Green), TF Group 2 (Red), TF Group 3 (Blue)). **E–G**. Gene process networks that are either activated (E, upregulated genes) or deactivated (F, downregulated genes) in MEFs transduced with the three TF module combinations as compared to negative control, were determined using the Thomson Reuters GeneGo MetaCore™ data meta-analysis tool (TF Group 1 (Green), TF Group 2 (Red), TF Group 3 (Blue)). Based on the list of significantly upregulated or downregulated genes each process network received a p-value indicating the statistical probability that the network is affected in the population of reprogrammed cells. The range of calculated p-values for each process network is graphically represented with a green to red color range (G). Activated networks: Lowest p-value: 3.16×10^−9^ (Green) and highest p-value: 6.53×10^−1^ (Red). Deactivated networks: Lowest p-value: 2.40×10^−40^ and highest p-value: 7.06×10^−1^ (Red). **H**. Graphical representation of hierarchical clustering analysis performed on the union of all of the significantly upregulated (Red, +2.69) or significantly downregulated genes (Blue, −2.69). **I–J**. Graphical representation of self-organizing map clustering analysis performed on the union of all of the significantly upregulated or significantly downregulated genes. We detected 22 unique self-organized gene groups for the upregulated genes (I) and 23 unique self-organized gene groups for the downregulated genes. Graphical representation of a normalized average gene expression pattern for each of the unique groups identified by self-organizing map clustering analysis for the three combinations of transcriptional modules (J) (Range: −1.5/Green to 1.5/Red).

To further ascertain the TFs’ overall effect on global gene expression, we performed microarray gene expression analysis on MEFs transduced with: TF Group 1 (*G_4_T_5_M_C_*), TF Group 2 (*G_4_T_5_M_C_M_1_S_3_*), TF Group 3 (*G_4_T_5_M_C_M_D_S_F_M_1_S_3_*), and negative control. The analysis was performed on the total population of MEFs. We first determined that the reprogrammed cells exhibited a variation in signal intensity for a number of probes (Figure 4A). We selected probe sets exhibiting a significant level of transcript upregulation or downregulation (p-value: <0.05, fold change<or >1.5) in each of the three groups as compared to the control (Figure 4B, Table S3). By comparing the three sets of probes, we identified a core set of commonly upregulated (1065) or downregulated (980) genes (Figure 4C–D, Table S4).

We implemented a pathway analysis tool to identify molecular pathways that were activated or inactivated in the cells undergoing epigenetic reprogramming. Previously described gene pathway networks received a calculated p-value based on the ratio of present-to-absent gene targets (Figure 4E–G). The cardio-inducing effect of TF group 3 was determined to be significantly higher as compared to that of TF group 1 or TF group 2 as it received significantly lower p-values for several cardiac-specific pathway networks. On the other hand, we detected a high level of alignment in all downregulated molecular pathways amongst all three TF groups. Notably downregulated pathways with the highest significance were related to some aspect of cell cycle control, indicating that cell division in general was significantly downregulated in the reprogrammed cells irrespective of the TF group applied and alluding to a common effect of GATA4, TBX5, and MEF2C.

Hierarchical clustering analysis performed on all probes exhibiting significant upregulation or downregulation in at least one of the three cell groups revealed particular probe set subgroups that differed in their level of gene expression (Figure 4H). We hypothesized that the differences recorded across the three cell groups were the result of a varying effect on activation or repression of downstream targets. To further differentiate the cardio-inducing effect between the three TF groups, we performed self-organizing map clustering analysis and ultimately organized the probe sets into unique clusters for upregulated or downregulated genes (Figure 4I–J, Table S5). We then performed pathway analysis on individual groups of clustered genes (Figure S8). Upregulated clusters #1–8 containing genes with a lower expression level in TF group 3 received the lowest p-values (highest significance) for network pathways including notch signaling, amyloid protein-based cell adhesion and ECM remodeling but no cardiac specific process networks. Upregulated clusters #15–22 containing genes with a higher expression level detected in TF group 3 received the lowest p-value for cardiac and muscle specific process networks including muscle contraction, skeletal muscle development, and cardiac development indicative of the significantly higher cardio-inducing potential of TF group 3 as compared to TF groups 1 and 2. Moreover, downregulated clusters 1–3, 6–7, 11, 12, and 15 containing genes with a lower expression level detected in cells transduced with TF group 3 received the lowest p-value for network pathways associated with cell cycle regulation. Downregulated clusters 13, 14, 18, 22, 23 containing genes with a higher expression level detected in cells transduced with TF group 3 received low p-values for network pathways including muscle and cardiac specific ones, further indicating the increased cardio-inducing potential of TF group 3.

Finally we evaluated the capacity of the three TF groups to induce cardiac cellular reprogramming in MEFs as compared to the effect described in a recent study reporting the successful *in vitro* reprogramming of cardiac fibroblasts into induced cardiomyocytes (iCM) [10]. We performed a network pathway analysis on genes selectively upregulated or downregulated in all three TF groups, heart control, and iCM (Figure S9A–C). Although TF group 1 is comprised of the same set of TF as the ones used in this study (main difference being that we used the human gene orthologs), we observed the closest correlation for upregulated genes between our TF group 3 and their TF group with both being closest to the heart control as compared to TF group 1 and 2. Moreover, for the downregulated genes the iCM cells were the furthest away from the heart control indicative of the lack of correlation between the two.

### Culture Conditions Enhance the Degree of Cytoskeletal Organization of Cardiac-specific Proteins

In addition to the evidence that short-term overexpression of certain TF module combinations led to transcriptional activation of cardiac-specific genes and expression of cardiac-specific proteins (although not properly organized), we examined whether the culture conditions would enhance this cardio-inducing effect. We screened three different media formulations including: a basic fibroblast growth medium (FGM), a cardiomyocyte growth medium (CGM), and a low serum growth medium (LSGM). We also examined whether extracellular matrix proteins may have a cardio-inducing effect by utilizing either gelatin or poly-l-lysine coated substrates [39]. Finally we examined the cardio-inducing effect of a JAK inhibitor which has been recently shown to enhance the cellular reprogramming capacity of mouse fibroblasts into cardiomyocytes [13].

**Figure 5:**
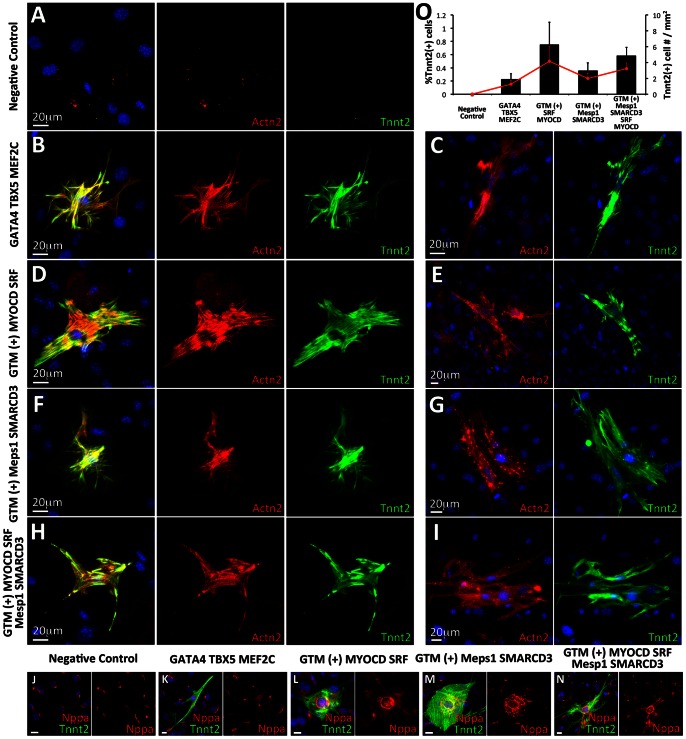
Reprogrammed MEFs express cardiac specific proteins and organize them in a cross-striated manner **A–I.** Induction of TF overexpression for 7 days in MEFs transduced with only FUW.M2rtTA or the four listed combinations of TF modules. The reprogrammed cells were cultured on gelatin-coated plastic in low serum growth medium. Using double-antibody immunofluorescence analysis (Actn2/Red, Tnnt2/Green) we detected cells expressing both cardiac proteins and organizing them in a cross-striated manner resembling cardiomyocytes (B, D, F, H). We detected significantly more double-positive cells in MEFs transduced with *G_4_T_5_M_C_M_D_S_F_*. For each of the transcriptional module combinations we also detected double-positive cells without any obvious cross-striated cytoskeletal organization (C, E, G, I). No Actn2, or Tnnt2 cross-striated expression was detected in the negative control cells. **J–N**. The Tnnt2 expressing cells also stained positive for the atrial protein marker Nppa. **O**. Quantification of the fraction of cells staining positive for the Tnnt2 cardiac protein (low serum growth medium) as compared to the total number of cells (black columns) and measurement of the fraction of Tnnt2-expressing cells per square millimeter (red line). Results are based on biological triplicates. Error bars represent calculated standard deviation. All four cell groups had a significant increase in the number of Tnnt2(+) cells as compared to the negative control group (P<0.01). Cells transduced with either *G_4_T_5_M_C_M_D_S_F_* (P<0.01), *G_4_T_5_M_C_M_D_S_F_M_1_S_3_* (P<0.05), *G_4_T_5_M_C_M_D_S_F_M_1_S_3_* (P<0.01) also had a significant increase in the number of Tnnt2(+) cells as compared to cells transduced with *G_4_T_5_M_C_*.

We did not detect cross-striated Actn2(+)/Tnnt2(+) cells following culture of any of the four induced cell populations in either of the two culture medium formulations containing high serum concentrations (FGM or CGM). We detected rare events where cells expressed the two proteins without however organizing them in a cross-striated manner. When culturing the cells in LSGM, however, we readily detected Actn2(+)/Tnnt2(+) cross-striated cells (Figure 5B, D, F, H). No Actn2(+)/Tnnt2(+) single or double-positive cells were detected in the negative control cell group (Figure 5A). We determined that Tnnt2 expression always coincided with Actn2 expression whereas Actn2 was detected in Tnnt2(**−**) cells. Tnnt2 expression was detected in all cross-striated cells indicating that the particular marker is more specific for the detection of cardiomyocytes. Cross-striations were detected in all four cell groups, although we detected a significantly higher percentage of these cells when using either *G_4_T_5_M_C_M_D_S_F_* (0.75±0.34%) or *G_4_T_5_M_C_M_D_S_F_M_1_S_3_* (0.58±0.13%) (Figure 5O). Additionally unlike the high level of cell proliferation observed when using the high serum media, very low levels of cell proliferation were detected when using LSGM and so both the density of the total number of cells as well as the density of the Tnnt2 positive cells was low even following 10 days of culture. We did not detect any significant cardio-inducing effect when culturing the cells on the two different extracellular matrix proteins. We did detect a small but significant cardio-inducing effect when using the JAK inhibitor leading to an average fold change of 1.73±0.32 (p-value: 0.006) in the amount of Tnnt2 positive cells detected. We also detected double positive Actn2(+)/Tnnt2(+) cells that did not organize the cytoskeletal proteins into cross-striated sarcomeres indicating that cellular reprogramming may have multiple stages of which not all are completed in target cells (Figure 5C, E, G, I). Over time we detected fewer cross-striated cells, and by day 30, the majority of double positive cells were not cross-striated indicating that the reprogrammed cells may require additional signaling for their long term survival and maintenance. Finally, we detected increased levels of Nppa protein expression in all four cell groups and in particular in the cells transduced with either *G_4_T_5_M_C_M_D_S_F_* or *G_4_T_5_M_C_M_D_S_F_M_1_S_3_* which closely correlates with the relative gene expression data reported earlier (Figure 5J–N).

### Electrophysiological Characterization of Reprogrammed Cells

**Figure 6:**
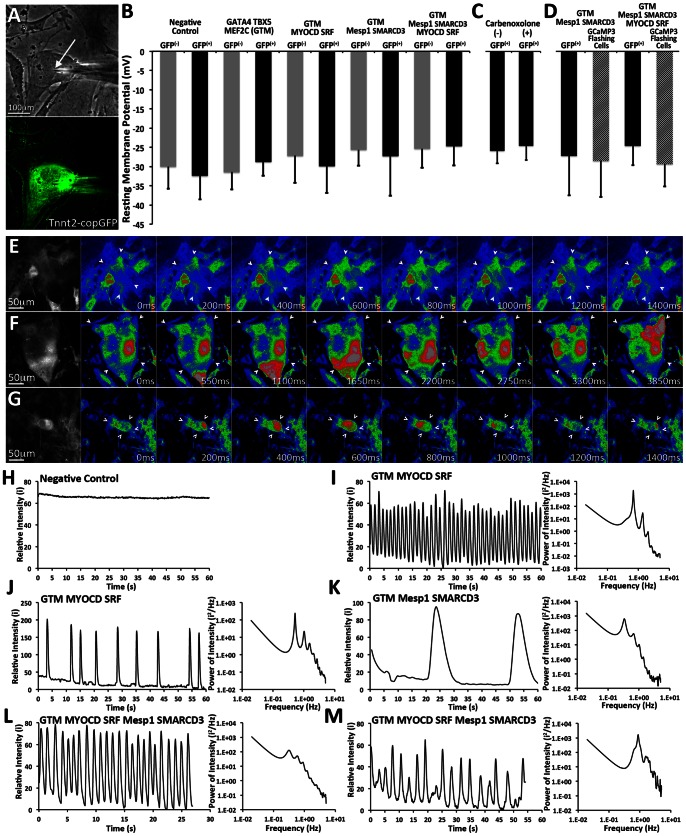
Electrophysiological characterization of reprogrammed cells. A–B. A–B. Recordings of RMP were performed using a sharp intracellular microelectrode (white arrow). RMP measurements were performed for either GFP(+) or GFP(**−**) cells in groups of MEFs transduced with either *G_4_T_5_M_C_* (n = 8 & 9), *G_4_T_5_M_C_M_D_S_F_* (n = 7 & 6), *G_4_T_5_M_C_M_1_S_3_* (n = 7 & 8), *G_4_T_5_M_C_M_D_S_F_M_1_S_3_* (n = 9 & 7), and negative control (n = 6 & 7). ANOVA was used to determine whether significant differences existed in the measured membrane resting potentials amongst the reported cell groups or between the GFP(+) and GFP(**−**) cell populations within each cell group. (One *for p-value <0.05, Two *for p-value <0.01). NRVMs as positive control (RMP: −75.78±3.14 mV, n = 8). Error bars represent calculated standard deviation. **C**. Sharp microelectrode recordings of RMP performed on GFP(+) cells in the presence or absence of 200 µM carbenoxolone, a gap junction uncoupler (n = 8 & 6). Error bars represent calculated standard deviation. **D**. MEFs were first transduced with either TNNT2.copGFP or TNNT2.GCaMP3 (conditional expression of fluorescent marker under the control of the TNNT2 promoter) and subsequently with the various combinations of TF modules. Sharp microelectrode recordings were performed on either GFP(+) cells or GCaMP3(+) cells that were also exhibiting regular GCaMP3 flashing (n = 8, 4, 7, & 9). Error bars represent calculated standard deviation. **E–G**. Serial frame images of individual cells GCaMP3 exhibiting flashing GCaMP3 signal (white arrows). Flashing GCaMP3 activity was detected in cells transduced with *G_4_T_5_M_C_M_D_S_F_* (E), *G_4_T_5_M_C_M_1_S_3_* (F), and *G_4_T_5_M_C_M_D_S_F_M_1_S_3_* (G). The rightmost panels show black and white images of the acquired flashing cells while the rest show color-coded RGB images based on signal intensity (Dark Blue Lowest intensity, Bright Red Highest Intensity). **H–M**. Plots (left panels) displaying the relative signal intensity for GCaMP3 over time in regions within cells exhibiting GCaMP3 flashing: Negative control (H), *G_4_T_5_M_C_M_D_S_F_* (I, J), *G_4_T_5_M_C_M_1_S_3_* (K), *G_4_T_5_M_C_M_D_S_F_M_1_S_3_* (L, M).

We set out to examine the electrophysiological characteristics of MEFs transduced with the various combinations of TF modules. We first performed sharp electrode intracellular recording of resting membrane potential (RMP) in MEFs containing the TNNT2-copGFP reporter vector (Figure 6A–B). NRVMs were used as positive control cells and their RMP was determined to be −75.78±3.14 mV (n = 8). Although the TNNT2-copGFP reporter vector was previously shown to have a GFP expression pattern that closely resembled the transcriptional activation of the endogenous *Tnnt2* gene locus (Figure 1D), we chose to perform the recordings on both GFP(+) and GFP(**−**) cells to account for any leakiness of GFP expression. We did not detect any significant differences in RMP amongst the GFP(+) or GFP(**−**) cells for all experimental groups or when comparing GFP(+) to GFP(**−**) cells (Figure 6B). We also recorded the RMP of GFP(+) transgenic mouse-derived MEFs (Myh6.eGFP) transduced with *G_4_T_5_M_C_M_D_S_F_M_1_S_3_* but their RMP (**−**31.15±10.46 mV, n = 12) was not significantly different from that recorded from negative control cells.

To investigate whether the recorded RMP in GFP(+) cells was influenced by potential intercellular coupling to surrounding cells via electrotonic loading [40], intracellular recordings were repeated on GFP(+) cells in the presence of 200 µM carbenoxolone, a gap junctional uncoupler [41]. Again no significant difference in RMP was observed in GFP(+) cells before (**−**28.7±3.7 mV, n = 9) or after (**−**27.3±4.2 mV, n = 6) the addition of carbenoxolone (Figure 6C).

To determine whether the reprogrammed cells cycle cytoplasmic Ca^2+^, we transduced MEFs with a lentivirus that constitutively expresses the genetically encoded calcium indicator GCaMP3 [24], [42]. Intracellular Ca^2+^ concentration fluctuations can be detected as Ca^2+^ binds to GCaMP3 and produces a transient increase in the intensity of its fluorescent signal.

Following, induction of TF module expression for 7 days we readily detected single cells or small groups of cells with a flashing GFP signal in MEFs transduced with either *G_4_T_5_M_C_M_D_S_F_* or *G_4_T_5_M_C_M_D_S_F_M_1_S_3_* (Figure 6E, 6G, Movie S2, Movie S4). We detected and recorded cells with fast and regular (Figure 6I, L), or slower and irregular Ca^2+^ transients (Figure 6J, M). Furthermore, calculating and plotting the GCaMP3 signal spectral intensity allowed us to study the multiple frequencies incorporated in the intensity signal as well as identify the primary frequency of that signal. The range of the primary frequencies was determined to be between 0.31 Hz and 0.89 Hz for the two transcription factor combinations. We also detected rare events of Ca^2+^ cycling in cells transduced with *G_4_T_5_M_C_M_1_S_3_* although the kinetics of Ca^2+^ release within these cells was very slow, and significantly different from that observed for the other two transcriptional module combinations (Figure 6K, Movie S3). We did not detect any changes in fluorescence intensity in the negative control cell population, indicating the absence of calcium cycling in those cells over an observation period of minutes (Figure 6H).

Finally, we recorded the resting membrane potential of reprogrammed flashing cells. To control for potential leaky activity of the reporter vectors, we utilized a reporter vector in which GCaMP3 expression was controlled by the cardiac Troponin promoter. MEFs were either transduced with TNNT2.copGFP or TNNT2.GCaMP3 and RMP measurements were recorded from either GFP(+) or GCaMP3(+) flashing cells (Figure 6D). No significant difference was detected in the membrane potential of the two cell groups when transduced with either of the transcriptional modules *G_4_T_5_M_C_M_1_S_3_* or *G_4_T_5_M_C_M_D_S_F_M_1_S_3_*.

## Discussion

Here we describe a systematic study to determine the capacity of ten transcription factors to induce a cardiomyocyte-like phenotype in cultures of MEFs. We demonstrate that TFs *M_D_S_F_* alone or in conjunction with *M_1_S_3_* significantly enhance the basal but indispensable cardio-inducing effect of *G_4_T_5_M_C_*. More specifically, when we overexpressed the two groups of either five or seven TF we detected: 1) TF-induced binding and activation of cardiac-specific promoter elements, 2) Expression of endogenous cardiac-specific genes including those encoding for cardiac cytoskeletal proteins and cardiac transcription factors. We also detected a mixed effect for calcium handling genes and cardiomyocyte ion-channel genes as some were upregulated whereas others were not changed. 3) Phenotypic cellular metamorphosis associated with cytoskeletal remodeling or reorganization and in particular detection of cross-striated cytoskeletal proteins, and 4) Calcium transient oscillations, although we did not detect significant changes in resting membrane potential or presence of contractile activity.

Interestingly we did not detect a cardio-inducing effect when transducing MEFs with either of the two TF pairs alone or together in the absence of *G_4_T_5_M_C_* signifying the importance of the latter as a necessary component in the cardio-inducing process [10]. Although this effect was evident when using lentivirally-delivered reporter vectors (Myl2.mCherry, TNNT2.copGFP), we detected a significant fraction of GFP(+) or mCherry(+) cells in the negative control, an observation likely related to the manner of reporter vector delivery, and indicative of a potential drawback in utilizing such an approach in detecting cellular reprogramming events accurately. However, when utilizing MEFs isolated from a transgenic mouse we readily detected a significant increase in the fraction of cells expressing GFP when using either only *M_D_S_F_* (1.60±0.12%) or in conjunction with *M_1_S_3_* (2.40±0.11%) as compared to the negative control (0.03±0.05%) or when just using *G_4_T_5_M_C_* (0.05±0.06%). Three groups studying the overexpression of Gata4, Tbx5, and Mef2c (mouse orthologs) in mouse cardiac fibroblasts, mouse tail-tip fibroblasts, or mouse embryonic fibroblasts have reported approximately 20% [10], 2.2% [14], and 0% [15] activation of a fluorescent marker controlled by the Myh6 promoter element. The reasons for this large discrepancy may include differences in experimental procedures, cell and virus preparation, or even timing of TF overexpression [14], [15]. In our study we utilized the human orthologs of the delivered genes (except for Mesp1), which may have affected the efficiency of cellular reprogramming.

We also detected a significant cardio-inducing enhancement when adding TF *M_D_S_F_* alone or with *M_1_S_3_* and both in conjunction with *G_4_T_5_M_C_* as determined by gene expression analysis. This effect was highly significant for *Actc1*, *Myh6*, *Myl2*, *Tnnt2*, *Casq2*, *Hcn4*, *Srf*, *Acta2*, *Nppa*, and *Myh11*. In the absence of *M_D_S_F_*, the previously detected cardio-inducing effect was eliminated when using only *G_4_T_5_M_C_* or in conjunction with *M_1_S_3_*. This may indicate that *M_D_S_F_* alone enhance the cardio-inducing effect of *G_4_T_5_M_C_* for the genes listed above whereas addition of *M_1_S_3_* alone does not significantly alter their expression levels. The strong cardio-inducing effect of *M_D_S_F_* in conjunction with *G_4_T_5_M_C_* may be due to the fact that both genes are active and necessary during embryonic cardiogenesis [43]–[45]. Interestingly addition of *M_D_S_F_* in conjunction with *G_4_T_5_M_C_* negated the upregulation recorded for the expression level of endogenous *Myocd,* which was only significantly upregulated when *M_1_S_3_* was used, whereas it was significantly downregulated in the other conditions. Additionally although overexpression of *G_4_T_5_M_C_* alone or with *M_1_S_3_* induced a significant downregulation in the relative expression levels of *Nkx2-5* and *Hop*, *M_D_S_F_* addition rescued this effect which is noteworthy since it has previously been shown that *Hop* expression is regulated by *Nkx2-5* and importantly it is an antagonist of *Srf* regulating the amount of cardiomyocytes in the developing heart [46], [47]. Future experiments may involve testing the cardio-inducing effect of *MYOCD* alone in the absence of *SRF* and more importantly in the presence of overexpressed *HOP*. It is also noteworthy to mention that endogenous *Tbx5*, and *Hand2* expression was significantly downregulated in all groups indicating that they are negatively regulated by the combination of *G_4_T_5_M_C_*. Based on our experimental observations *G_4_T_5_M_C_* expression alone had a generally weak cardio-inducing effect, although we detected a significant upregulation of *Tnnt2* expression as previously reported [15]. Also, the weak or absent activation of transcriptional expression of smooth muscle or endothelial markers suggests that the cardiac reprogramming of fibroblasts does not proceed through the formation of a multipotent cardiac progenitor cell [10], [19]. Finally, our gene expression data suggested that both ventricular and atrial cardiomyocyte-like subtypes were present in the cells undergoing epigenetic reprogramming as evident by expression of Myl2, Myl7 and Nppa. In our future studies we plan to further characterize these cellular subtypes using electrophysiogical characterization.

The enhanced cardio-inducing effect of the TF pair *M_D_S_F_* in conjunction with *G_4_T_5_M_C_* was particularly evident when we performed global gene expression analysis. Cardiac development and muscle-function gene process networks identified based on global upregulated gene expression received significantly lower p-values for cells which were also transduced with *M_D_S_F_*. We detected activation of gene process networks that are associated with cell adhesion, which may explain why the MEFs could not be easily enzymatically dissociated following induction of TF expression. On the other hand, when analyzing downregulated genes the global effect on gene expression was homogeneous and highly aligned in all three TF groups used, indicative of a role of the common denominator transcription factor module: *G_4_T_5_M_C_*. More importantly the gene process networks that were determined to be significantly downregulated are associated with aspects of the cell cycle. This finding is aligned with our observation that we detected a lack of proliferation, which is to be expected if the MEFs were being reprogrammed into cardiomyocytes [48]. It has recently been demonstrated that the number of cell divisions during the derivation of induced pluripotent stem cells is a fundamental variable driving epigenetic reprogramming to pluripotency with every cell being capable of eventually being reprogrammed into an iPS cell [49]. This inherently may create a significant limitation for the potential of increasing the efficiency of cardiac cell reprogramming, as the TF used in this study inhibit cell proliferation and may in turn limit their access to the binding sites in the genome. This may also explain the fact that we detected so many immaturely reprogrammed cells lacking cytoskeletal organization or proper electrophysiological properties. Finally, using global gene expression analysis we determined that MEFs transduced with *G_4_T_5_M_C_M_D_S_F_M_1_S_3_* were more similar with the induced cardiomyocytes derived using Gata4, Tbx5, and Mef2c [10], an observation aligned with the FACS data indicating potential differences in the cell source used, the method of gene delivery, or the human versus mouse gene orthologs. In the future we plan to include within this analysis gene expression profiles of unique cardiac resident cell subtypes including ventricular or atrial cardiomyocytes, pacemaker cells, cardiac fibroblasts, smooth muscle, and endothelial cells. Such an analysis would better elucidate the gene expression signature of the reprogrammed cells.

Addition of small molecules significantly enhanced the fraction of cells expressing cardiac proteins. Valproic acid, a histone deacetylase inhibitor, previously shown to significantly improve the efficiency of pluripotency induction [36], [44], provided a two-fold increase of the cardio-inducing effect in our experiments. Furthermore, use of the JAK inhibitor which was previously shown to have pro-cardiogenic activity [13], [50], also significantly enhanced the cardio-inducing effect of the delivered TF. Although addition of these two small molecules significantly increased the fraction of cells expressing cardiac specific proteins, organization of these proteins in a cross-striated manner was only depended on cells being cultured in the low-serum growth medium. This suggests that the means of action of these two molecules is to increase TF accessibility to their DNA binding sites, or to prime the cells for reprogramming, thus increasing the probability that the target cells initiate cardiac protein expression. On the other hand, culture of the cells undergoing reprogramming in low serum growth medium seems to be the determinant of cytoskeletal maturation. In general culture of MEFs under low serum conditions significantly decreases their proliferative capacity and since the TF used in this study cause a significant downregulation of cell cycling it is possible that the low serum culture conditions enhance this effect. Further studies are necessary to define the effect of the culture conditions on initiation of reprogramming and maturation of the desired cells. Additionally, we would like to optimize the culture conditions with the ultimate goal of significantly improving the completeness and efficiency of reprogramming fibroblasts into cardiomyocytes [51]. Such an approach should include optimizing the time window during which the TF are being overexpressed. It should also include determining the ideal TF stoichiometry which has been previously shown to influence both the state and the properties of the derived cells [52].

We did not detect a significant hyperpolarization of the RMP in MEFs transduced with any of the TF combinations up to 7 days following induction of expression. Previously the RMP for wild-type mouse cardiomyocytes was reported to be approximately −56 to −63 mV [53], [54] and a similar RMP (**−**57 mV) was recently reported in a study describing the reprogramming of mouse fibroblasts into cardiomyocytes [10]. Moreover, hyperpolarization of RMP to a level of that expected to be recorded in cardiomyocytes was only detected following long-term cell culture which may indicate that the proteins responsible for RMP hyperpolarization are only expressed in later stages of reprogramming. However in our study long-term cell culture caused loss of cross-striated sarcomeres and selective proliferation of non-transduced fibroblasts. This is consistent with our observation that we did not detect significant upregulation in genes conferring electrophysiological function including *Atp2a1*, *Atp2a2*, *Cacna1a*, *Kcnj2*, *Kcnj3*, *Kcnk1*, *Pln*, and *Scn5a*. The recorded RMPs in both the GFP(+) and GFP(**−**) MEFs more closely resembled those observed in other unexcitable cells including mesenchymal stem cells (**−**19 to **−**35 mV) [55], [56], skeletal myoblasts (**−**26 to **−**44 mV) [57], [58], and cardiac fibroblasts (**−**20 to **−**37 mV) [59], [60].

On the other hand, we readily detected increased incidence of intracellular Ca^2+^ oscillations using the genetically encoded calcium indicator GCaMP3 [24], [42], particularly in MEFs transduced with *M_D_S_F_* alone or with *M_1_S_3_* in conjunction with *G_4_T_5_M_C_*. This observation is in agreement with recent reports describing the detection of spontaneous Ca^2+^ oscillations of variable frequency in reprogrammed mouse fibroblasts [10], [15]. Although we detected Ca^2+^ oscillations in cells transduced with only *G_4_T_5_M_C_M_1_S_3_*, the intracellular calcium waves were significantly slower as compared to those transduced with *G_4_T_5_M_C_M_D_S_F_M_1_S_3_*. Overall, based on the depolarized resting potential that would preclude Ca^2+^ flux through membrane-bound, voltage-dependent Ca^2+^ channels, the observed intracellular Ca^2+^ transients likely originated from cyclical oscillations of Ca^2+^ levels in intracellular calcium stores.

A reemerging observation evident throughout our study is the strong enhancement of the cardio-inductive effect of *G_4_T_5_M_C_* achieved by the addition of *M_D_S_F_*. Myocardin is a strong transcriptional coactivator expressed in cardiomyocytes and smooth muscle cells during postnatal development, and along with Mkl1 and Mkl2 it associates with Srf which binds on CArG DNA motifs activating transcription [61]. Forced Myocd overexpression has been shown to activate expression of Acta2, Tagln, and Myh11 [43], [62]. Importantly, Myocd is required for the maintenance of heart function by maintaining sarcomeric organization and intercalated disc structures, and promoting cardiomyocyte survival. Although Myocd expression has not been detected in cardiac fibroblasts thus far, its cofactor Mlk1 is indeed expressed and contributes to the induction of the myofibroblast phenotype following myocardial infarction injury [63]. Two recent reports demonstrated that following myocardial infarction delivery of Gata4, Mef2c, and Tbx5, or GATA4, HAND2, MEF2C, and TBX5 in the injured myocardium successfully reprogrammed cardiac fibroblasts into cardiomyocytes [16], [17]. In these studies the authors report that the *in vivo* derived cells were more fully reprogrammed, more closely resembled host cardiomyocytes, and that TF overexpression induced a functional improvement that was greater to that predicted based on the *in vitro* reprogramming efficiency of the same TF combination. We hypothesize that, based on our observation when using the *M_D_S_F_* transcriptional module, that the reported *in vivo* effects may be the result of transduction and cellular reprogramming of activated myofibroblasts present in the infarcted region, which although may be lacking Myocd expression, have activated the Srf/Mlk1 transcriptional pathways. Additional experiments will need to be performed with fibroblasts and activated myofibroblasts to test whether the activation process itself would enhance the cardiac reprogramming efficiency.

In conclusion, here we describe a detailed study to determine the capacity of a core set of transcription factors to induce cellular reprogramming of primary fibroblasts into cardiomyocytes. We demonstrate that MYOCD and SRF alone or in conjunction with Mesp1 and SMARCD3 significantly enhance the cardio-inducing effect of GATA4, TBX5, and MEF2C. We also show that derivation of cardiomyocyte-like cells containing well-organized cross-striated sarcomeres is highly dependent on the culture conditions used during reprogramming. The reprogrammed cells develop the capacity to cycle intracellular Ca^2+^, although we do not detect significant membrane hyperpolarization or spontaneous contractions. It is evident that the complex genetic networks active during embryonic cardiac development can also induce cardiac cell reprogramming of non-cardiac cell types although this process is currently inefficient and poorly understood. Our work sheds light into the role of some of the main genetic regulators participating in this process.

## Supporting Information

Figure S1 **Expression validation of each of the transcription factors delivered using the inducible lentivirus-based delivery system.** **A**. Qualitative RT.PCR analysis was used to detect expression of each of the transcription factors delivered in the various combinations of transcriptional modules. MEFs were first transduced with only a lentivirus allowing the constitutive expression of M2rtTA. Following cell expansion cells were passaged and further transduced over two days with the various combinations of transcriptional modules. Induction of expression of the transcription factors was achieved by the addition of doxycycline in the culture medium. Following three days of induction of expression, qualitative RT.PCR analysis was used to detect expression of each transcription factor (23 cycles for Gapdh and 28 cycles for the other genes). **B**. The capacity of the “tet-on” inducible expression system was tested in MEFs using a control reporter vector where GFP was cloned in the 3′ end of the inducible promoter element. No GFP expression was detected in MEFs transduced with FUW.M2rtTA and FU.tet.on.GFP in the absence of doxycycline in the culture medium. Doxycycline addition triggered robust GFP expression as early as 1 day post induction initiation and lasting for at least 7 days.
(TIF)

Figure S2 **Screening analysis for the cardio-inducing effect of the TF modules in the murine NIH3T3 cell line and MEFs.** **A**. NIH3T3 transduced with the Myh6.eGFP reporter vector and M2rtTA were subsequently transduced with 14 different combinations of TF modules including the positive control. Following induction of expression for 7 days the fraction of cells expressing GFP was determined by fluorescent activated cell sorting (FACS). Negative control cells were either transduced with only FUW.M2rtTA or FUW.M2rtTA and Myh6.eGFP. Positive control cells were transduced with FU.tet.on.GFP. NIH3T3 cells were readily transduced with the lentivirus as evidenced by the percentage of GFP(+) cells (99.8±0.04%) in the positive control sample (FU-tet-on-GFP). A fraction of the negative control cells, which were transduced with only the reporter vector (Myh6.eGFP) while receiving no additional TF, were expressing GFP (1.26±0.10%), which is indicative of either the leaky nature of this particular promoter element or the non-specific integration of the lentiviral DNA fragment in transcriptionally active genomic loci. Following TF module transduction and induction of expression for 7 days the most significant upregulation in the fraction of GFP(+) cells was detected in MEFs transduced with either *M_D_S_F_M_1_S_3_* (2.99±0.06%) or *G_4_T_5_M_C_M_D_S_F_M_1_S_3_* (1.82±0.10%). A significant decrease in the fraction of GFP(+) cells was observed when using the N_5_H_1_H_2_ TF module, which may indicate binding and repression of the particular Myh6 promoter element by some or all of the included TF. The largest decrease in GFP(+) cells was recorded when using *M_D_S_F_M_1_S_3_N_5_H_1_H_2_* (0.05±0.04%). (One *for p-value <0.05, Two *for p-value <0.01). Error bars represent calculated standard deviation. **B–E**. Measuring the relative gene expression levels of Myh6, Myl2 and Tnnt2 using quantitative RT.PCR analysis in NIH3T3 or primary MEFs transduced with the various listed combinations of transcriptional modules. Using gene expression analysis we recorded a significant upregulation in endogenous Myh6 transcription in cells transduced with all the TF module combinations, although the most significant was recorded in cells transduced with either *G_4_T_5_M_C_M_D_S_F_*, or *G_4_T_5_M_C_M_D_S_F_M_1_S_3_*. The effect of the combinatorial TF module overexpression on the endogenous level of *Myh6* transcription did not correlate with the effect measured using the Myh6.eGFP reporter vector. In the case of *Myl2* transcription, the only TF module that induced a large and significant upregulation effect was *M_1_S_3_*. That effect was abolished when *M_1_S_3_* was co-expressed with any of the other three TF modules. Finally, a much clearer and significant transcriptional effect was established when measuring the relative expression level of the endogenous *Tnnt2* which suggested a strong effect of the *G_4_T_5_M_C_* TF module in its activation. A strong *Tnnt2* expression as a result of *G_4_T_5_M_C_* was consistent in both the NIH3T3 and primary MEFs. The effect of *G_4_T_5_M_C_* on the level of *Tnnt2* expression was more profound when using NIH3T3 (100–1000X), which may indicate an increased epigenetic accessibility of the overexpressed TF in the immortalized cell line as compared to primary MEFs. Error bars represent calculated standard deviation. (One *for p-value <0.05, Two *for p-value <0.01). Error bars represent calculated standard deviation.
(TIF)

Figure S3 **Positive control immunofluorescence staining.** Primary cultures of isolated neonatal rat ventricular myocytes (NRVMs) were stained using antibodies raised against the Actn2, Tnnt2, Myh6, Myl2, Gja1, and Acta2 cardiac proteins. Following rat ventricular tissue digestion, cardiac fibroblasts were removed following two cycles of pre-plating and subsequently NRVMs were plated on fibronectin-coated cell culture plates. Fixation and immunofluorescent analysis was performed 7 days following initial plating and culture. Cells were spontaneously contracting in synchrony prior to fixation.
(TIF)

Figure S4 **Valproic acid enhances the cardio-inducing effect of the transcriptional modules in MEFs.** Primary MEFs were transduced with either *G_4_T_5_M_C_* or *G_4_T_5_M_C_M_D_S_F_* and transcription factor expression was induced for 7 days in the presence or absence of valproic acid (0.5 mM). Immunofluorescence was used to assay the additive cardio-inducing effect of valproic acid addition with antibodies against either Actn2 or Tnnt2.
(TIF)

Figure S5 **Relative gene expression level analysis for Myh6, Myh7, Myl2, Myl7, Nppa, and Tnnt2 in populations of sorted and subsequently cultured GFP(+) MEFs, and GFP(−) MEFs transduced with** ***G_4_T_5_M_C_*** **and the Myh6.eGFP reporter vector.** Negative control cells were only transduced with FUW.M2rtTA. Error bars represent calculated standard deviation.
(TIF)

Figure S6 **Confirmation of GFP expression in a cardiac-specific manner in spontaneously contracting cardiomyocytes derived from differentiated induced pluripotent stem cells derived from primary transgenic MEFs (Myh6.eGFP.Myh6.PAC). A**. We used a previously described [18] lentiviral vector (FU.tet.on.OSKM) to successfully reprogram primary MEFs isolated from the transgenic mice. Colonies of undifferentiated iPS cells were readily detectable within 10 days following induction of expression of Oct4, Sox2, Klf4, and Myc. Colonies were mechanically picked, enzymatically dissociated, and passaged on feeder layers of mitotically-inactivated primary MEFs. **B**. We used the previously described [19] hanging droplet technique to differentiate the derived iPSCs. Within 7 days of differentiation we readily detected spontaneously contracting population of GFP-expressing cardiomyocytes. This allowed us to confirm that the transgenic MEFs retain their capacity to express GFP in a cardiac specific manner even when undergoing a cycle of reprogramming into undifferentiated iPSC and then being differentiated again.
(TIF)

Figure S7 **Gene expression analysis of primary MEFs transduced with three combinations of transcriptional modules.** MEFs were transduced with FUW.M2rtTA and *G_4_T_5_M_C_*, or *G_4_T_5_M_C_M_1_S_3_*, or *G_4_T_5_M_C_M_D_S_F_M_1_S_3_*. We isolated RNA on 7 days post induction of transcription factor expression. Using quantitative RT.PCR and custom-designed TaqMan Low Density Array plates we measured the relative gene expression levels normalizing to negative control MEFs (FUW.M2rtTA only). (One *for p-value <0.05, Two *for p-value <0.01). Error bars represent calculated standard deviation.
(TIF)

Figure S8 **Genes belonging to groups of gene clusters showing similar patterns were further analyzed using the Thomson Reuters GeneGo MetaCore™ data meta-analysis tool: clusters 1–8 (upregulated), clusters 15–22 (upregulated), clusters 1–3 & 6–7 (downregulated), clusters 11, 12, 15 (downregulated), clusters 13, 14, 18, 22, 23 (downregulated).** Process networks were determined for each of the groups based on a p-value score with the most significant having the lowest p-value.
(TIF)

Figure S9 **Microarray gene expression analysis performed on populations of transdifferentiated MEFs, Heart Control, and induced cardiomyocytes (iCMs).** **A–C**. Gene process networks that are either activated (A, upregulated genes) or deactivated (B, downregulated genes) in MEFs transduced with the three transcriptional module combinations as compared to negative control, heart positive control as compared to MEFs negative control, and iCMs as compared to cardiac fibroblasts negative control, were determined using the Thomson Reuters GeneGo MetaCore™ data meta-analysis tool (TF Group 1 Green, TF Group 2 Red, TF Group 3 Blue, Heart Control Black, iCMs Doted line). Based on the list of significantly upregulated or downregulated genes each process network received a p-value indicating the statistical probability that the network is affected in the population of transdifferentiated cells, or heart control. The range of calculated p-values for each process network is graphically represented with a green to red color range (C). Activated networks: Lowest p-value: 4.12×10^−18^ (Green) and highest p-value: 9.81×10^−1^ (Red). Deactivated networks: Lowest p-value: 5.17×10^−41^ and highest p-value: 1 (Red). **D–E**. Principal component analysis was performed on the p-values calculated for each of the process networks for either significantly upregulated (D), or significantly downregulated genes (E). Correlation circle plots are graphically represented for each of the three transcription factor groups, the iCMs, and the heart.
(TIF)

Table S1 **Lists of primers and Taqman probe IDs used for qualitative and quantitative RT.PCR analysis as well as the ones used to design the custom-made TLDA plates.**
(XLSX)

Table S2 **Calculated fold change for genes that are significantly upregulated or downregulated when comparing the transcriptome of heart (positive control) to that of MEFs (negative control).** The genes in this list are the same presented in Figure 2.
(XLSX)

Table S3 **List of significantly upregulated and downregulated genes determined when comparing each of the transdifferentiated cell groups (TF Group 1** ***G_4_T_5_M_C_*** **, TF Group 2** ***G_4_T_5_M_C_M_1_S_3_*** **, TF Group 3** ***G_4_T_5_M_C_M_D_S_F_M_1_S_3_*** **) to MEFs (negative control).**
(XLSX)

Table S4 **List of common and significantly upregulated or downregulated genes determined when comparing each of the transdifferentiated cell groups (TF Group 1** ***G_4_T_5_M_C_*** **, TF Group 2** ***G_4_T_5_M_C_M_1_S_3_*** **, TF Group 3** ***G_4_T_5_M_C_M_D_S_F_M_1_S_3_*** **) to MEFs (negative control).**
(XLSX)

Table S5 **Results of self-organized map clustering of significantly upregulated (22 clusters) and downregulated (23 clusters) genes when comparing each of the transdifferentiated cell groups (TF Group 1** ***G_4_T_5_M_C_*** **, TF Group 2** ***G_4_T_5_M_C_M_1_S_3_*** **, TF Group 3** ***G_4_T_5_M_C_M_D_S_F_M_1_S_3_*** **) to MEFs (negative control).**
(XLSX)

Movie S1 **Induced pluripotent stem cells derived from transgenic MEFs (Myh6.eGFP.Myh6.PAC) using a single inducible polycystronic lentiviral vector containing all four reprogramming factors (Oct4, Sox2, Klf4, Myc) were differentiated using the hanging droplet technique.** Regions containing spontaneously contracting cardiomyocytes were readily detected as early as 7 days post initiation of differentiation. GFP expression was specific to the spontaneously contracting cells.
(MP4)

Movie S2 **MEFs were initially transduced with FUW.M2rtTA and a lentivirus permitting the constitutive expression of the genetically encoded calcium indicator GCaMP3, and subsequently with the various combinations of the described transcriptional modules.** Cells exhibiting flashing or transient increase in GFP intensity were detected in the group transduced with *G_4_T_5_M_C_M_D_S_F_*. Further analysis was performed using FIJI and an RGB lookup table was applied to visualize the range of signal intensity (Dark blue Least intense, Bright red Most intense): *G_4_T_5_M_C_M_D_S_F_* (1∶05).
(MP4)

Movie S3 **MEFs were initially transduced with FUW.M2rtTA and a lentivirus permitting the constitutive expression of the genetically encoded calcium indicator GCaMP3, and subsequently with the various combinations of the described transcriptional modules.** Cells exhibiting flashing or transient increase in GFP intensity were detected in the group transduced with *G_4_T_5_M_C_M_D_S_F_ G_4_T_5_M_C_M_1_S_3_*. Further analysis was performed using FIJI and an RGB lookup table was applied to visualize the range of signal intensity (Dark blue Least intense, Bright red Most intense): *G_4_T_5_M_C_M_1_S_3_* (0∶51).
(MP4)

Movie S4 **MEFs were initially transduced with FUW.M2rtTA and a lentivirus permitting the constitutive expression of the genetically encoded calcium indicator GCaMP3, and subsequently with the various combinations of the described transcriptional modules.** Cells exhibiting flashing or transient increase in GFP intensity were detected in the group transduced with *G_4_T_5_M_C_M_D_S_F_M_1_S_3_*. Further analysis was performed using FIJI and an RGB lookup table was applied to visualize the range of signal intensity (Dark blue Least intense, Bright red Most intense): *G_4_T_5_M_C_M_D_S_F_M_1_S_3_* (0∶26).
(MP4)

## References

[1] Murry CE, Reinecke H, Pabon LM (2006) Regeneration gaps: observations on stem cells and cardiac repair. Journal of the American College of Cardiology 47: 1777–1785. 16682301 10.1016/j.jacc.2006.02.002

[2] Laflamme MA, Murry CE (2011) Heart regeneration. Nature 473: 326–335. 21593865 10.1038/nature10147 PMC4091722

[3] Burridge PW, Keller G, Gold JD, Wu JC (2012) Production of de novo cardiomyocytes: human pluripotent stem cell differentiation and direct reprogramming. Cell stem cell 10: 16–28. 22226352 10.1016/j.stem.2011.12.013 PMC3255078

[4] Maltsev VA, Rohwedel J, Hescheler J, Wobus AM (1993) Embryonic stem cells differentiate in vitro into cardiomyocytes representing sinusnodal, atrial and ventricular cell types. Mechanisms of development 44: 41–50. 8155574 10.1016/0925-4773(93)90015-p

[5] Kehat I, Kenyagin-Karsenti D, Snir M, Segev H, Amit M, et al (2001) Human embryonic stem cells can differentiate into myocytes with structural and functional properties of cardiomyocytes. The Journal of clinical investigation 108: 407–414. 11489934 10.1172/JCI12131 PMC209357

[6] Takahashi K, Yamanaka S (2006) Induction of pluripotent stem cells from mouse embryonic and adult fibroblast cultures by defined factors. Cell 126: 663–676. 16904174 10.1016/j.cell.2006.07.024

[7] Takahashi K, Tanabe K, Ohnuki M, Narita M, Ichisaka T, et al (2007) Induction of pluripotent stem cells from adult human fibroblasts by defined factors. Cell 131: 861–872. 18035408 10.1016/j.cell.2007.11.019

[8] Mauritz C, Schwanke K, Reppel M, Neef S, Katsirntaki K, et al (2008) Generation of functional murine cardiac myocytes from induced pluripotent stem cells. Circulation 118: 507–517. 18625890 10.1161/CIRCULATIONAHA.108.778795

[9] Zhang J, Wilson GF, Soerens AG, Koonce CH, Yu J, et al (2009) Functional cardiomyocytes derived from human induced pluripotent stem cells. Circulation research 104: e30–41. 19213953 10.1161/CIRCRESAHA.108.192237 PMC2741334

[10] Ieda M, Fu JD, Delgado-Olguin P, Vedantham V, Hayashi Y, et al (2010) Direct reprogramming of fibroblasts into functional cardiomyocytes by defined factors. Cell 142: 375–386. 20691899 10.1016/j.cell.2010.07.002 PMC2919844

[11] Szabo E, Rampalli S, Risueno RM, Schnerch A, Mitchell R, et al (2010) Direct conversion of human fibroblasts to multilineage blood progenitors. Nature 468: 521–526. 21057492 10.1038/nature09591

[12] Olson EN (2006) Gene regulatory networks in the evolution and development of the heart. Science 313: 1922–1927. 17008524 10.1126/science.1132292 PMC4459601

[13] Jayawardena TM, Egemnazarov B, Finch EA, Zhang L, Payne JA, et al (2012) MicroRNA-mediated in vitro and in vivo direct reprogramming of cardiac fibroblasts to cardiomyocytes. Circulation research 110: 1465–1473. 22539765 10.1161/CIRCRESAHA.112.269035 PMC3380624

[14] Protze S, Khattak S, Poulet C, Lindemann D, Tanaka EM, et al (2012) A new approach to transcription factor screening for reprogramming of fibroblasts to cardiomyocyte-like cells. Journal of molecular and cellular cardiology 53: 323–332. 22575762 10.1016/j.yjmcc.2012.04.010

[15] Chen JX, Krane M, Deutsch MA, Wang L, Rav-Acha M, et al (2012) Inefficient reprogramming of fibroblasts into cardiomyocytes using Gata4, Mef2c, and Tbx5. Circulation research 111: 50–55. 22581928 10.1161/CIRCRESAHA.112.270264 PMC3390172

[16] Qian L, Huang Y, Spencer CI, Foley A, Vedantham V, et al (2012) In vivo reprogramming of murine cardiac fibroblasts into induced cardiomyocytes. Nature 485: 593–598. 22522929 10.1038/nature11044 PMC3369107

[17] Song K, Nam YJ, Luo X, Qi X, Tan W, et al (2012) Heart repair by reprogramming non-myocytes with cardiac transcription factors. Nature 485: 599–604. 22660318 10.1038/nature11139 PMC3367390

[18] Carey BW, Markoulaki S, Hanna J, Saha K, Gao Q, et al (2009) Reprogramming of murine and human somatic cells using a single polycistronic vector. Proceedings of the National Academy of Sciences of the United States of America 106: 157–162. 19109433 10.1073/pnas.0811426106 PMC2629226

[19] Christoforou N, Miller RA, Hill CM, Jie CC, McCallion AS, et al (2008) Mouse ES cell-derived cardiac precursor cells are multipotent and facilitate identification of novel cardiac genes. The Journal of clinical investigation 118: 894–903. 18246200 10.1172/JCI33942 PMC2214848

[20] Maherali N, Ahfeldt T, Rigamonti A, Utikal J, Cowan C, et al (2008) A high-efficiency system for the generation and study of human induced pluripotent stem cells. Cell stem cell 3: 340–345. 18786420 10.1016/j.stem.2008.08.003 PMC3987901

[21] Lois C, Hong EJ, Pease S, Brown EJ, Baltimore D (2002) Germline transmission and tissue-specific expression of transgenes delivered by lentiviral vectors. Science 295: 868–872. 11786607 10.1126/science.1067081

[22] Barth AS, Kizana E, Smith RR, Terrovitis J, Dong P, et al (2008) Lentiviral vectors bearing the cardiac promoter of the Na+-Ca2+ exchanger report cardiogenic differentiation in stem cells. Molecular therapy : the journal of the American Society of Gene Therapy 16: 957–964. 18388932 10.1038/mt.2008.30 PMC2717010

[23] Kita-Matsuo H, Barcova M, Prigozhina N, Salomonis N, Wei K, et al (2009) Lentiviral vectors and protocols for creation of stable hESC lines for fluorescent tracking and drug resistance selection of cardiomyocytes. PloS one 4: e5046. 19352491 10.1371/journal.pone.0005046 PMC2662416

[24] Tian L, Hires SA, Mao T, Huber D, Chiappe ME, et al (2009) Imaging neural activity in worms, flies and mice with improved GCaMP calcium indicators. Nature methods 6: 875–881. 19898485 10.1038/nmeth.1398 PMC2858873

[25] Hockemeyer D, Soldner F, Cook EG, Gao Q, Mitalipova M, et al (2008) A drug-inducible system for direct reprogramming of human somatic cells to pluripotency. Cell stem cell 3: 346–353. 18786421 10.1016/j.stem.2008.08.014 PMC4097107

[26] Schindelin J, Arganda-Carreras I, Frise E, Kaynig V, Longair M, et al (2012) Fiji: an open-source platform for biological-image analysis. Nature methods 9: 676–682. 22743772 10.1038/nmeth.2019 PMC3855844

[27] Welch P (1967) The use of fast Fourier transform for the estimation of power spectra: A method based on time averaging over short, modified periodograms. Audio and Electroacoustics, IEEE Transactions on 15: 70–73.

[28] Takeuchi JK, Bruneau BG (2009) Directed transdifferentiation of mouse mesoderm to heart tissue by defined factors. Nature 459: 708–711. 19396158 10.1038/nature08039 PMC2728356

[29] Lindsley RC, Gill JG, Murphy TL, Langer EM, Cai M, et al (2008) Mesp1 coordinately regulates cardiovascular fate restriction and epithelial-mesenchymal transition in differentiating ESCs. Cell stem cell 3: 55–68. 18593559 10.1016/j.stem.2008.04.004 PMC2497439

[30] Bondue A, Lapouge G, Paulissen C, Semeraro C, Iacovino M, et al (2008) Mesp1 acts as a master regulator of multipotent cardiovascular progenitor specification. Cell stem cell 3: 69–84. 18593560 10.1016/j.stem.2008.06.009

[31] Small EM, Warkman AS, Wang DZ, Sutherland LB, Olson EN, et al (2005) Myocardin is sufficient and necessary for cardiac gene expression in Xenopus. Development 132: 987–997. 15673566 10.1242/dev.01684

[32] Wang Z, Wang DZ, Pipes GC, Olson EN (2003) Myocardin is a master regulator of smooth muscle gene expression. Proceedings of the National Academy of Sciences of the United States of America 100: 7129–7134. 12756293 10.1073/pnas.1232341100 PMC165841

[33] Miano JM, Ramanan N, Georger MA, de Mesy Bentley KL, Emerson RL, et al (2004) Restricted inactivation of serum response factor to the cardiovascular system. Proceedings of the National Academy of Sciences of the United States of America 101: 17132–17137. 15569937 10.1073/pnas.0406041101 PMC535359

[34] McFadden DG, Barbosa AC, Richardson JA, Schneider MD, Srivastava D, et al (2005) The Hand1 and Hand2 transcription factors regulate expansion of the embryonic cardiac ventricles in a gene dosage-dependent manner. Development 132: 189–201. 15576406 10.1242/dev.01562

[35] Tanaka M, Chen Z, Bartunkova S, Yamasaki N, Izumo S (1999) The cardiac homeobox gene Csx/Nkx2.5 lies genetically upstream of multiple genes essential for heart development. Development 126: 1269–1280. 10021345 10.1242/dev.126.6.1269

[36] Huangfu D, Osafune K, Maehr R, Guo W, Eijkelenboom A, et al (2008) Induction of pluripotent stem cells from primary human fibroblasts with only Oct4 and Sox2. Nature biotechnology 26: 1269–1275. 10.1038/nbt.1502 18849973

[37] Subramaniam A, Gulick J, Neumann J, Knotts S, Robbins J (1993) Transgenic analysis of the thyroid-responsive elements in the alpha-cardiac myosin heavy chain gene promoter. J Biol Chem 268: 4331–4336. 8440718

[38] Kattman SJ, Huber TL, Keller GM (2006) Multipotent flk-1+ cardiovascular progenitor cells give rise to the cardiomyocyte, endothelial, and vascular smooth muscle lineages. Developmental cell 11: 723–732. 17084363 10.1016/j.devcel.2006.10.002

[39] Addis RC, Hsu FC, Wright RL, Dichter MA, Coulter DA, et al (2011) Efficient conversion of astrocytes to functional midbrain dopaminergic neurons using a single polycistronic vector. PloS one 6: e28719. 22174877 10.1371/journal.pone.0028719 PMC3235158

[40] McSpadden LC, Kirkton RD, Bursac N (2009) Electrotonic loading of anisotropic cardiac monolayers by unexcitable cells depends on connexin type and expression level. American journal of physiology Cell physiology 297: C339–351. 19494239 10.1152/ajpcell.00024.2009 PMC2724091

[41] de GrootJR, Veenstra T, Verkerk AO, Wilders R, Smits JP, et al (2003) Conduction slowing by the gap junctional uncoupler carbenoxolone. Cardiovascular research 60: 288–297. 14613858 10.1016/j.cardiores.2003.07.004

[42] Tallini YN, Ohkura M, Choi BR, Ji G, Imoto K, et al (2006) Imaging cellular signals in the heart in vivo: Cardiac expression of the high-signal Ca2+ indicator GCaMP2. Proceedings of the National Academy of Sciences of the United States of America 103: 4753–4758. 16537386 10.1073/pnas.0509378103 PMC1450242

[43] Wang D, Chang PS, Wang Z, Sutherland L, Richardson JA, et al (2001) Activation of cardiac gene expression by myocardin, a transcriptional cofactor for serum response factor. Cell 105: 851–862. 11439182 10.1016/s0092-8674(01)00404-4

[44] Huang J, Min Lu M, Cheng L, Yuan LJ, Zhu X, et al (2009) Myocardin is required for cardiomyocyte survival and maintenance of heart function. Proceedings of the National Academy of Sciences of the United States of America 106: 18734–18739. 19850880 10.1073/pnas.0910749106 PMC2773995

[45] Niu Z, Iyer D, Conway SJ, Martin JF, Ivey K, et al (2008) Serum response factor orchestrates nascent sarcomerogenesis and silences the biomineralization gene program in the heart. Proceedings of the National Academy of Sciences of the United States of America 105: 17824–17829. 19004760 10.1073/pnas.0805491105 PMC2584699

[46] Shin CH, Liu ZP, Passier R, Zhang CL, Wang DZ, et al (2002) Modulation of cardiac growth and development by HOP, an unusual homeodomain protein. Cell 110: 725–735. 12297046 10.1016/s0092-8674(02)00933-9

[47] Chen F, Kook H, Milewski R, Gitler AD, Lu MM, et al (2002) Hop is an unusual homeobox gene that modulates cardiac development. Cell 110: 713–723. 12297045 10.1016/s0092-8674(02)00932-7

[48] Soonpaa MH, Field LJ (1998) Survey of studies examining mammalian cardiomyocyte DNA synthesis. Circulation research 83: 15–26. 9670914 10.1161/01.res.83.1.15

[49] Hanna J, Saha K, Pando B, van Zon J, Lengner CJ, et al (2009) Direct cell reprogramming is a stochastic process amenable to acceleration. Nature 462: 595–601. 19898493 10.1038/nature08592 PMC2789972

[50] Efe JA, Hilcove S, Kim J, Zhou H, Ouyang K, et al (2011) Conversion of mouse fibroblasts into cardiomyocytes using a direct reprogramming strategy. Nature cell biology 13: 215–222. 21278734 10.1038/ncb2164

[51] Chen J, Liu J, Chen Y, Yang J, Liu H, et al (2011) Rational optimization of reprogramming culture conditions for the generation of induced pluripotent stem cells with ultra-high efficiency and fast kinetics. Cell research 21: 884–894. 21445094 10.1038/cr.2011.51 PMC3203703

[52] Carey BW, Markoulaki S, Hanna JH, Faddah DA, Buganim Y, et al (2011) Reprogramming factor stoichiometry influences the epigenetic state and biological properties of induced pluripotent stem cells. Cell stem cell 9: 588–598. 22136932 10.1016/j.stem.2011.11.003

[53] Ke Q, Xiao YF, Bradbury JA, Graves JP, Degraff LM, et al (2007) Electrophysiological properties of cardiomyocytes isolated from CYP2J2 transgenic mice. Molecular pharmacology 72: 1063–1073. 17652182 10.1124/mol.107.035881 PMC2243182

[54] Xiao YF, TenBroek EM, Wilhelm JJ, Iaizzo PA, Sigg DC (2006) Electrophysiological characterization of murine HL-5 atrial cardiomyocytes. American journal of physiology Cell physiology 291: C407–416. 16571870 10.1152/ajpcell.00020.2006

[55] Heubach JF, Graf EM, Leutheuser J, Bock M, Balana B, et al (2004) Electrophysiological properties of human mesenchymal stem cells. The Journal of physiology 554: 659–672. 14578475 10.1113/jphysiol.2003.055806 PMC1664789

[56] Pijnappels DA, Schalij MJ, Ramkisoensing AA, van Tuyn J, de Vries AA, et al (2008) Forced alignment of mesenchymal stem cells undergoing cardiomyogenic differentiation affects functional integration with cardiomyocyte cultures. Circulation research 103: 167–176. 18556577 10.1161/CIRCRESAHA.108.176131

[57] Bernheim L, Liu JH, Hamann M, Haenggeli CA, Fischer-Lougheed J, et al (1996) Contribution of a non-inactivating potassium current to the resting membrane potential of fusion-competent human myoblasts. The Journal of physiology 493 (Pt 1): 129–141. 10.1113/jphysiol.1996.sp021369 PMC1158955 8735699

[58] Iannaccone ST, Li KX, Sperelakis N (1987) Transmembrane electrical characteristics of cultured human skeletal muscle cells. Journal of cellular physiology 133: 409–413. 3680398 10.1002/jcp.1041330230

[59] Chilton L, Ohya S, Freed D, George E, Drobic V, et al (2005) K+ currents regulate the resting membrane potential, proliferation, and contractile responses in ventricular fibroblasts and myofibroblasts. American journal of physiology Heart and circulatory physiology 288: H2931–2939. 15653752 10.1152/ajpheart.01220.2004

[60] Rook MB, van Ginneken AC, de Jonge B, el Aoumari A, Gros D, et al (1992) Differences in gap junction channels between cardiac myocytes, fibroblasts, and heterologous pairs. The American journal of physiology 263: C959–977. 1279981 10.1152/ajpcell.1992.263.5.C959

[61] Huang J, Min Lu M, Cheng L, Yuan LJ, Zhu X, et al (2009) Myocardin is required for cardiomyocyte survival and maintenance of heart function. Proc Natl Acad Sci U S A 106: 18734–18739. 19850880 10.1073/pnas.0910749106 PMC2773995

[62] Xing W, Zhang TC, Cao D, Wang Z, Antos CL, et al (2006) Myocardin induces cardiomyocyte hypertrophy. Circ Res 98: 1089–1097. 16556869 10.1161/01.RES.0000218781.23144.3e

[63] Small EM, Thatcher JE, Sutherland LB, Kinoshita H, Gerard RD, et al (2010) Myocardin-related transcription factor-a controls myofibroblast activation and fibrosis in response to myocardial infarction. Circ Res 107: 294–304. 20558820 10.1161/CIRCRESAHA.110.223172 PMC2921870

