# Supplementary figures and images for: Transcription Factors MYOCD, SRF, Mesp1 and SMARCD3 Enhance the Cardio-Inducing Effect of GATA4, TBX5, and MEF2C during Direct Cellular Reprogramming
Source: PLoS One. 2013 May 21;8(5):e63577. doi: 10.1371/journal.pone.0063577 (PMC3660533; doi:10.1371/journal.pone.0063577)

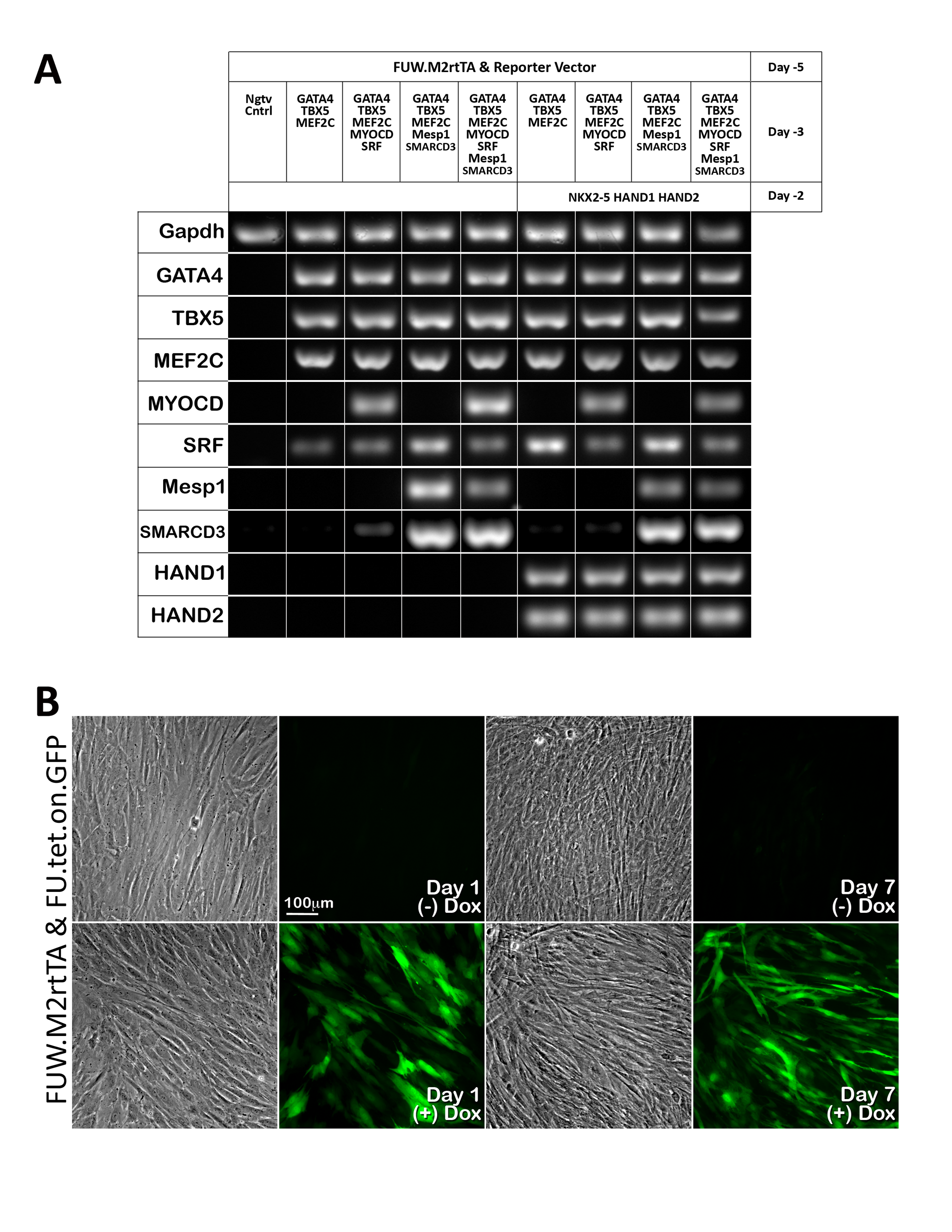

Supplement: Figure S1 — Expression validation of each of the transcription factors delivered using the inducible lentivirus-based delivery system. A. Qualitative RT.PCR analysis was used to detect expression of each of the transcription factors delivered in the various combinations of transcriptional modules. MEFs were first transduced with only a lentivirus allowing the constitutive expression of M2rtTA. Following cell expansion cells were passaged and further transduced over two days with the various combinations of transcriptional modules. Induction of expression of the transcription factors was achieved by the addition of doxycycline in the culture medium. Following three days of induction of expression, qualitative RT.PCR analysis was used to detect expression of each transcription factor (23 cycles for Gapdh and 28 cycles for the other genes). B. The capacity of the “tet-on” inducible expression system was tested in MEFs using a control reporter vector where GFP was cloned in the 3′ end of the inducible promoter element. No GFP expression was detected in MEFs transduced with FUW.M2rtTA and FU.tet.on.GFP in the absence of doxycycline in the culture medium. Doxycycline addition triggered robust GFP expression as early as 1 day post induction initiation and lasting for at least 7 days. (TIF) [file pone.0063577.s001.tif]

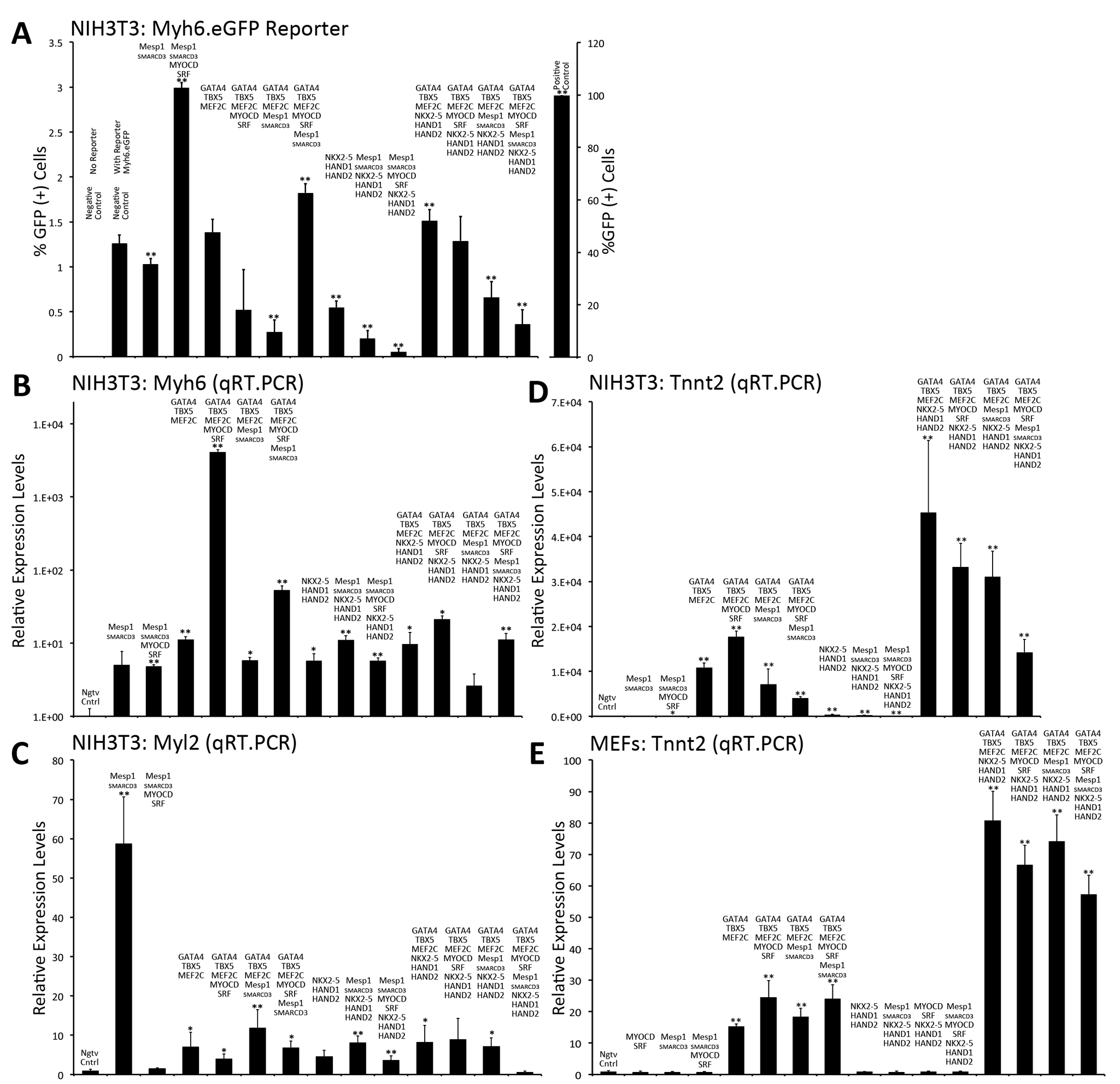

Supplement: Figure S2 — Screening analysis for the cardio-inducing effect of the TF modules in the murine NIH3T3 cell line and MEFs. A. NIH3T3 transduced with the Myh6.eGFP reporter vector and M2rtTA were subsequently transduced with 14 different combinations of TF modules including the positive control. Following induction of expression for 7 days the fraction of cells expressing GFP was determined by fluorescent activated cell sorting (FACS). Negative control cells were either transduced with only FUW.M2rtTA or FUW.M2rtTA and Myh6.eGFP. Positive control cells were transduced with FU.tet.on.GFP. NIH3T3 cells were readily transduced with the lentivirus as evidenced by the percentage of GFP(+) cells (99.8±0.04%) in the positive control sample (FU-tet-on-GFP). A fraction of the negative control cells, which were transduced with only the reporter vector (Myh6.eGFP) while receiving no additional TF, were expressing GFP (1.26±0.10%), which is indicative of either the leaky nature of this particular promoter element or the non-specific integration of the lentiviral DNA fragment in transcriptionally active genomic loci. Following TF module transduction and induction of expression for 7 days the most significant upregulation in the fraction of GFP(+) cells was detected in MEFs transduced with either MDSFM1S3 (2.99±0.06%) or G4T5MCMDSFM1S3 (1.82±0.10%). A significant decrease in the fraction of GFP(+) cells was observed when using the N5H1H2 TF module, which may indicate binding and repression of the particular Myh6 promoter element by some or all of the included TF. The largest decrease in GFP(+) cells was recorded when using MDSFM1S3N5H1H2 (0.05±0.04%). (One *for p-value <0.05, Two *for p-value <0.01). Error bars represent calculated standard deviation. B–E. Measuring the relative gene expression levels of Myh6, Myl2 and Tnnt2 using quantitative RT.PCR analysis in NIH3T3 or primary MEFs transduced with the various listed combinations of transcriptional modules. Using gene expression analysis we r [file pone.0063577.s002.tif]

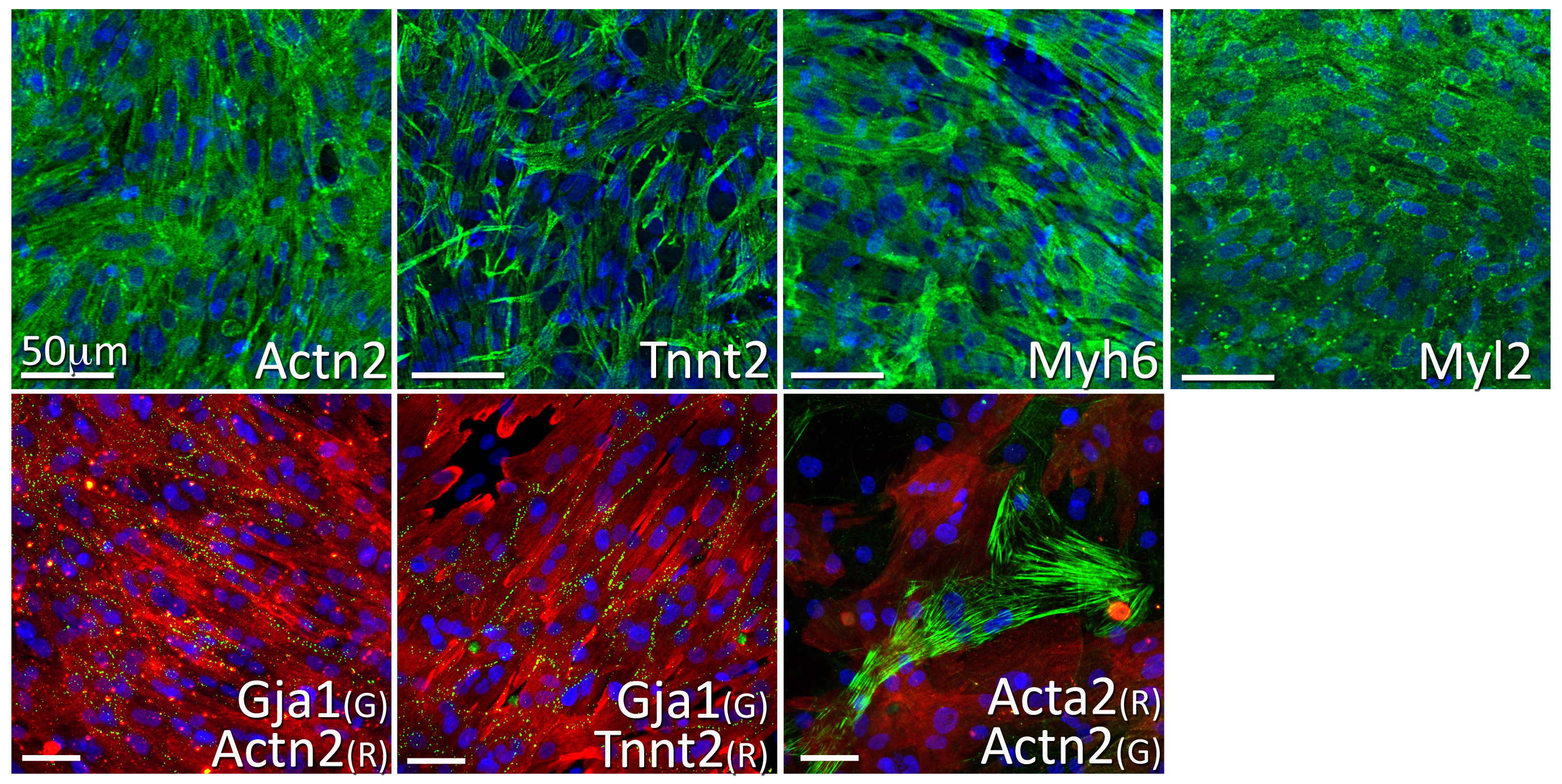

Supplement: Figure S3 — Positive control immunofluorescence staining. Primary cultures of isolated neonatal rat ventricular myocytes (NRVMs) were stained using antibodies raised against the Actn2, Tnnt2, Myh6, Myl2, Gja1, and Acta2 cardiac proteins. Following rat ventricular tissue digestion, cardiac fibroblasts were removed following two cycles of pre-plating and subsequently NRVMs were plated on fibronectin-coated cell culture plates. Fixation and immunofluorescent analysis was performed 7 days following initial plating and culture. Cells were spontaneously contracting in synchrony prior to fixation. (TIF) [file pone.0063577.s003.tif]

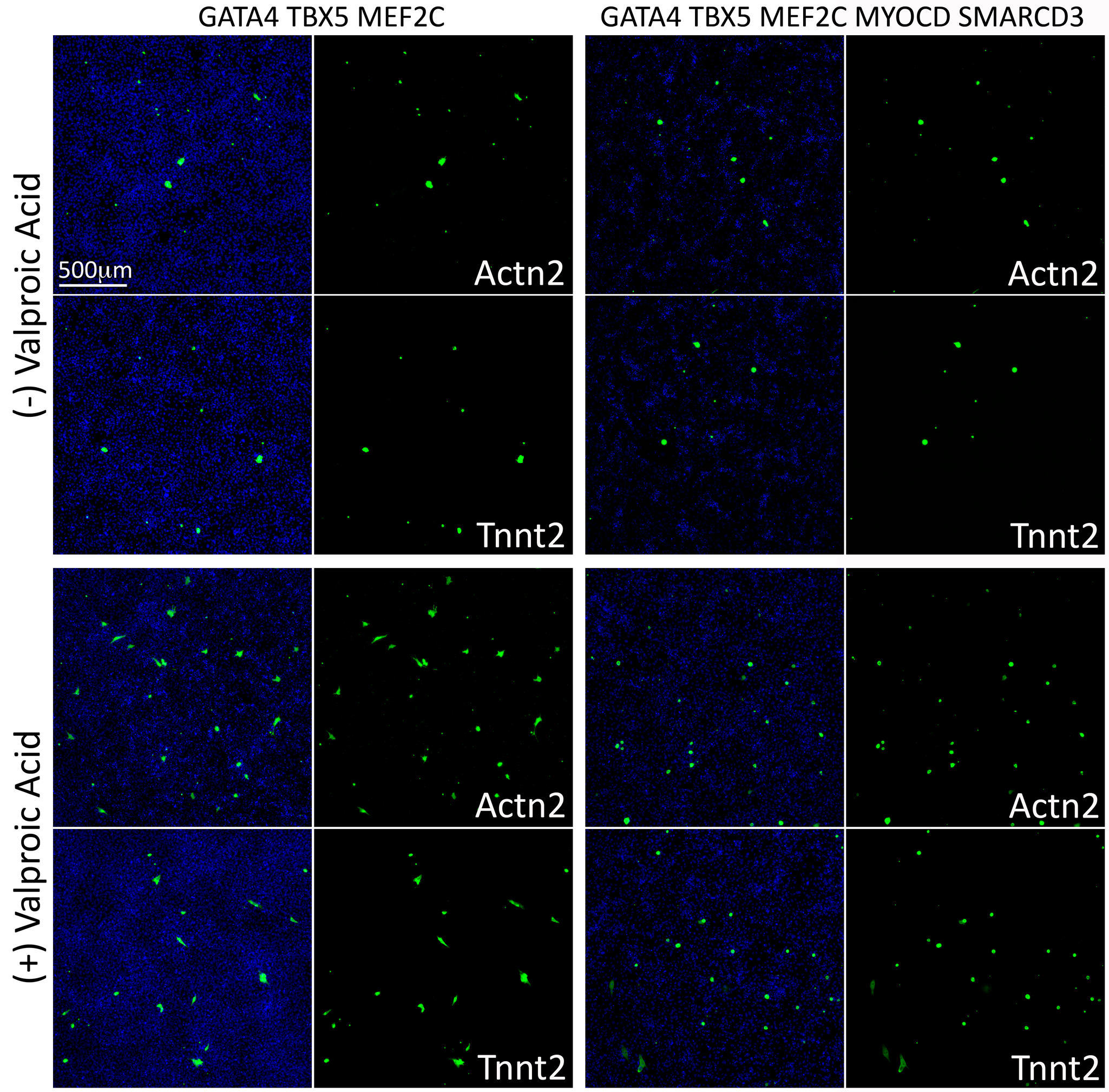

Supplement: Figure S4 — Valproic acid enhances the cardio-inducing effect of the transcriptional modules in MEFs. Primary MEFs were transduced with either G4T5MC or G4T5MCMDSF and transcription factor expression was induced for 7 days in the presence or absence of valproic acid (0.5 mM). Immunofluorescence was used to assay the additive cardio-inducing effect of valproic acid addition with antibodies against either Actn2 or Tnnt2. (TIF) [file pone.0063577.s004.tif]

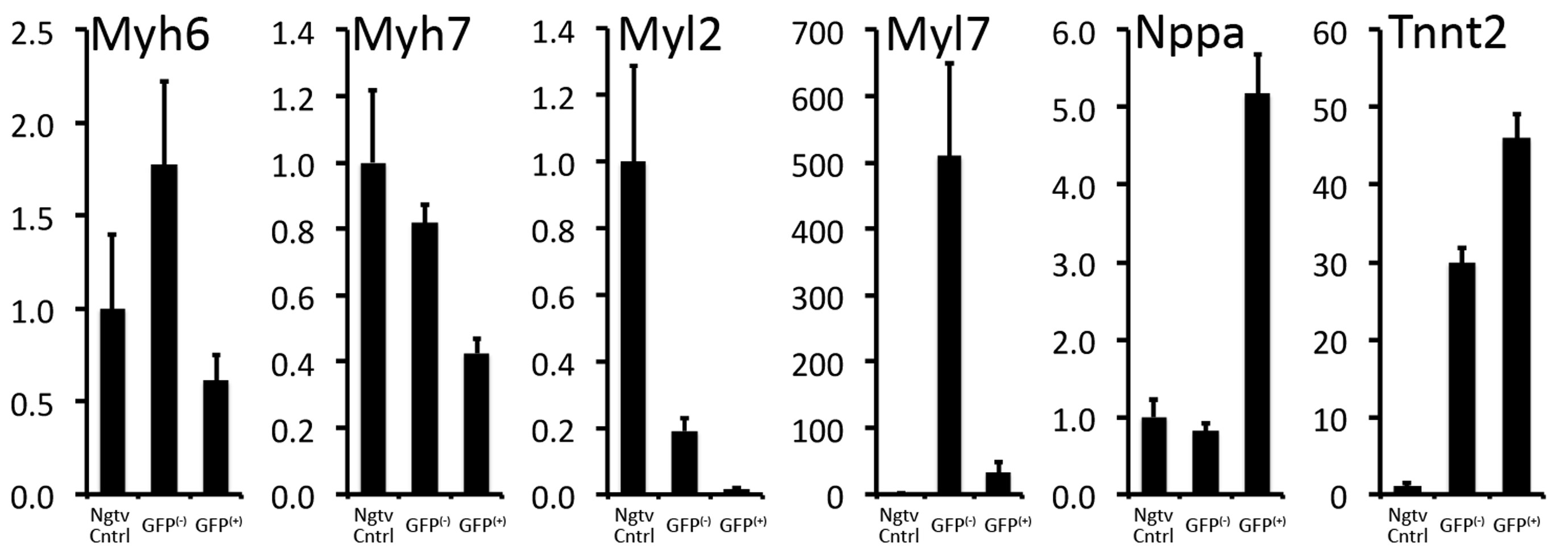

Supplement: Figure S5 — Relative gene expression level analysis for Myh6, Myh7, Myl2, Myl7, Nppa, and Tnnt2 in populations of sorted and subsequently cultured GFP(+) MEFs, and GFP(−) MEFs transduced with G4T5MC and the Myh6.eGFP reporter vector. Negative control cells were only transduced with FUW.M2rtTA. Error bars represent calculated standard deviation. (TIF) [file pone.0063577.s005.tif]

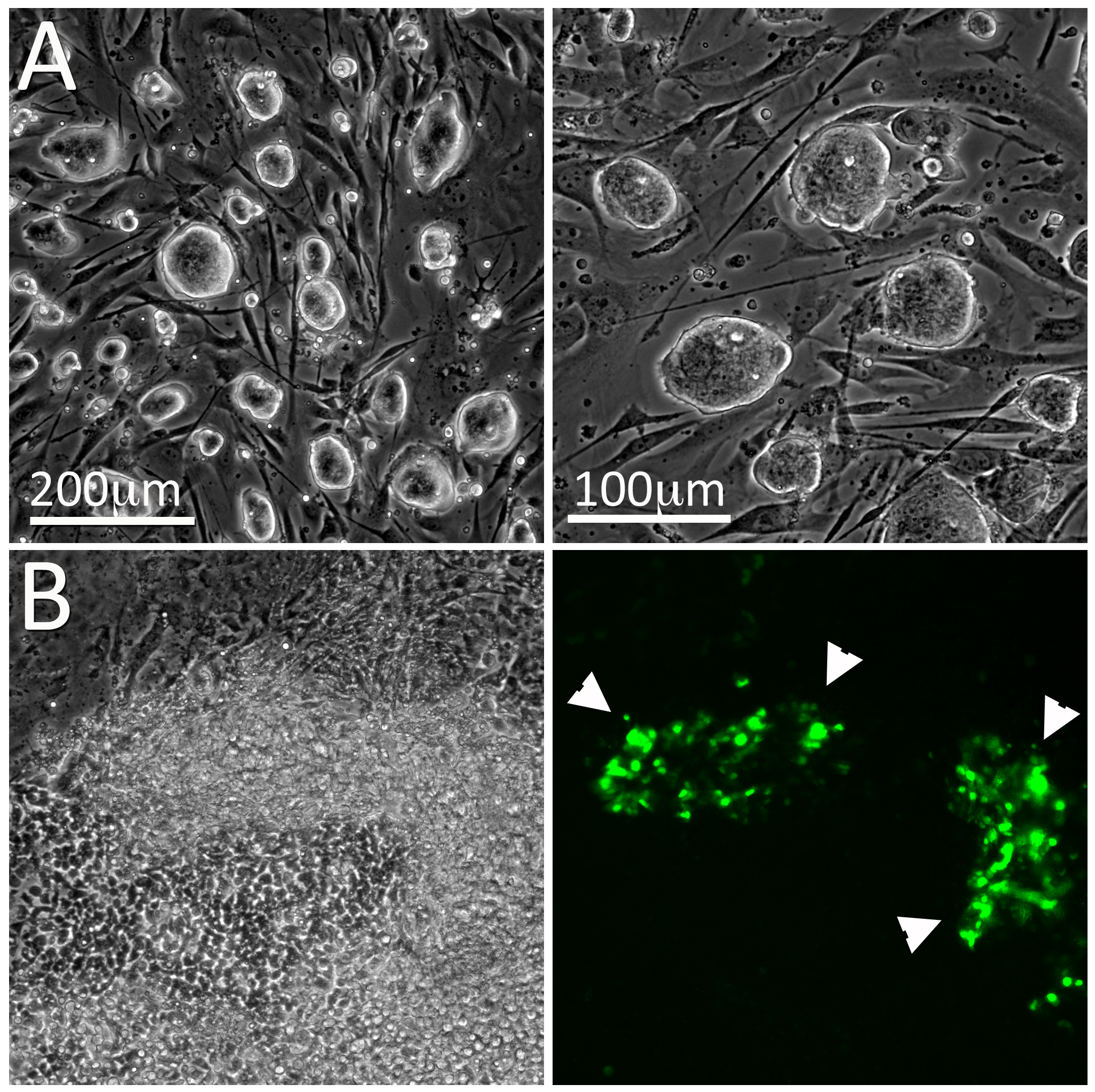

Supplement: Figure S6 — Confirmation of GFP expression in a cardiac-specific manner in spontaneously contracting cardiomyocytes derived from differentiated induced pluripotent stem cells derived from primary transgenic MEFs (Myh6.eGFP.Myh6.PAC). A. We used a previously described [18] lentiviral vector (FU.tet.on.OSKM) to successfully reprogram primary MEFs isolated from the transgenic mice. Colonies of undifferentiated iPS cells were readily detectable within 10 days following induction of expression of Oct4, Sox2, Klf4, and Myc. Colonies were mechanically picked, enzymatically dissociated, and passaged on feeder layers of mitotically-inactivated primary MEFs. B. We used the previously described [19] hanging droplet technique to differentiate the derived iPSCs. Within 7 days of differentiation we readily detected spontaneously contracting population of GFP-expressing cardiomyocytes. This allowed us to confirm that the transgenic MEFs retain their capacity to express GFP in a cardiac specific manner even when undergoing a cycle of reprogramming into undifferentiated iPSC and then being differentiated again. (TIF) [file pone.0063577.s006.tif]

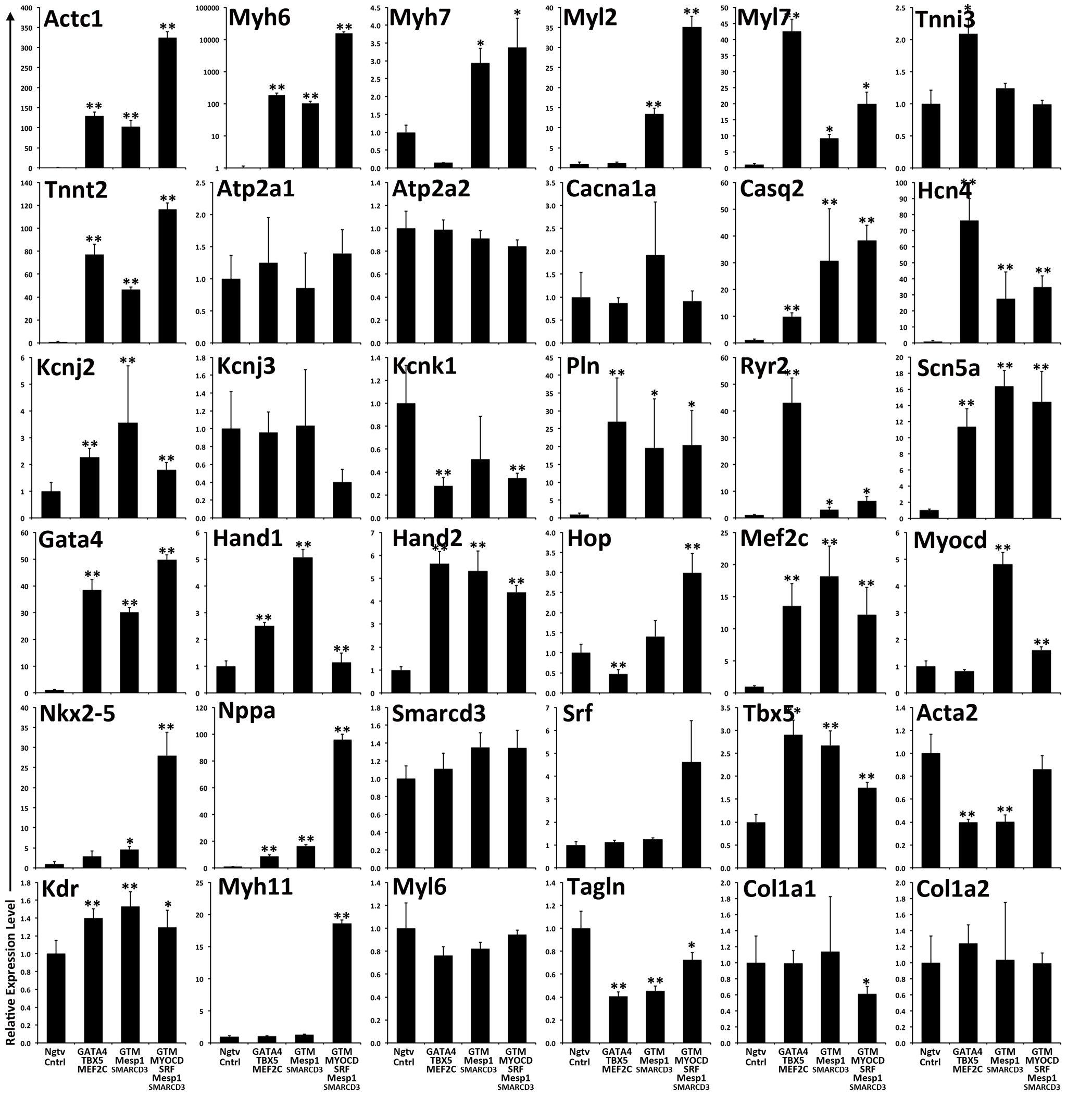

Supplement: Figure S7 — Gene expression analysis of primary MEFs transduced with three combinations of transcriptional modules. MEFs were transduced with FUW.M2rtTA and G4T5MC, or G4T5MCM1S3, or G4T5MCMDSFM1S3. We isolated RNA on 7 days post induction of transcription factor expression. Using quantitative RT.PCR and custom-designed TaqMan Low Density Array plates we measured the relative gene expression levels normalizing to negative control MEFs (FUW.M2rtTA only). (One *for p-value <0.05, Two *for p-value <0.01). Error bars represent calculated standard deviation. (TIF) [file pone.0063577.s007.tif]

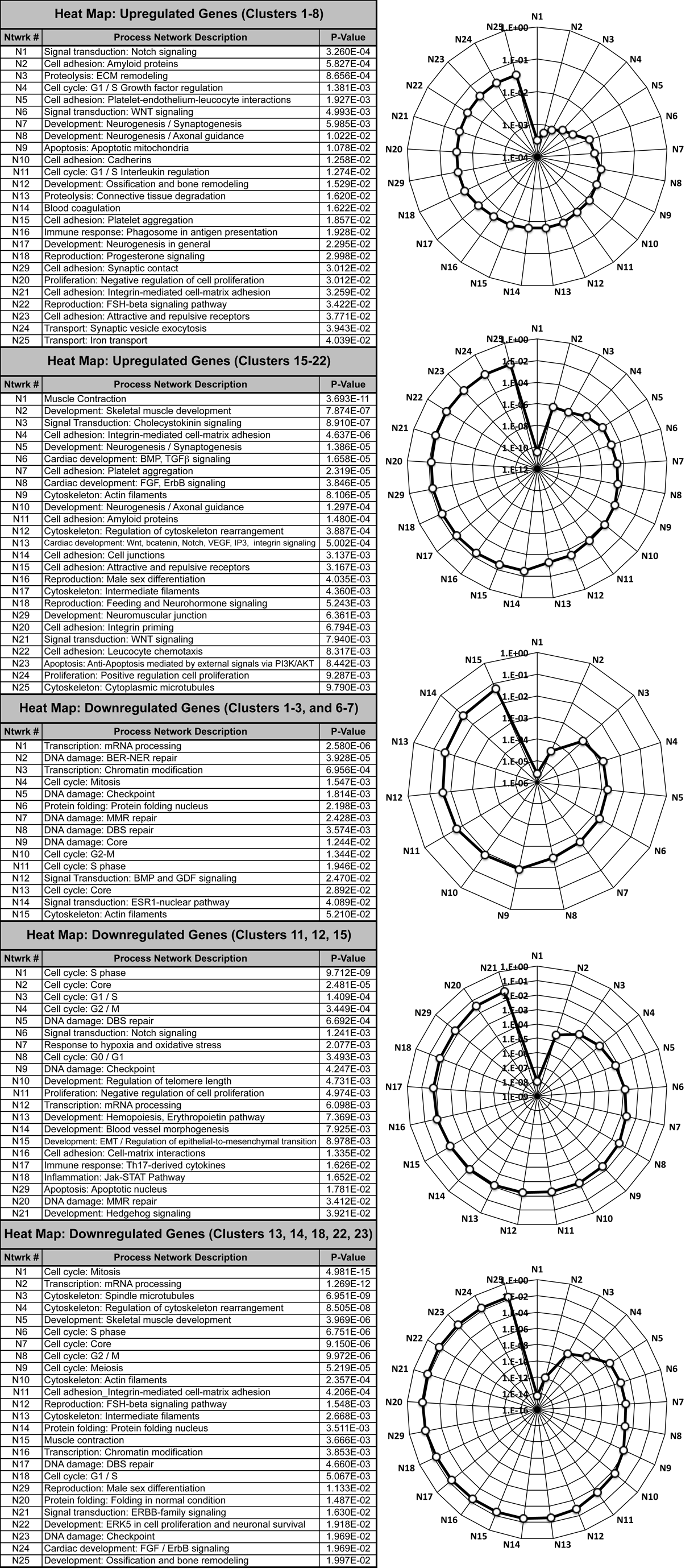

Supplement: Figure S8 — Genes belonging to groups of gene clusters showing similar patterns were further analyzed using the Thomson Reuters GeneGo MetaCore™ data meta-analysis tool: clusters 1–8 (upregulated), clusters 15–22 (upregulated), clusters 1–3 & 6–7 (downregulated), clusters 11, 12, 15 (downregulated), clusters 13, 14, 18, 22, 23 (downregulated). Process networks were determined for each of the groups based on a p-value score with the most significant having the lowest p-value. (TIF) [file pone.0063577.s008.tif]

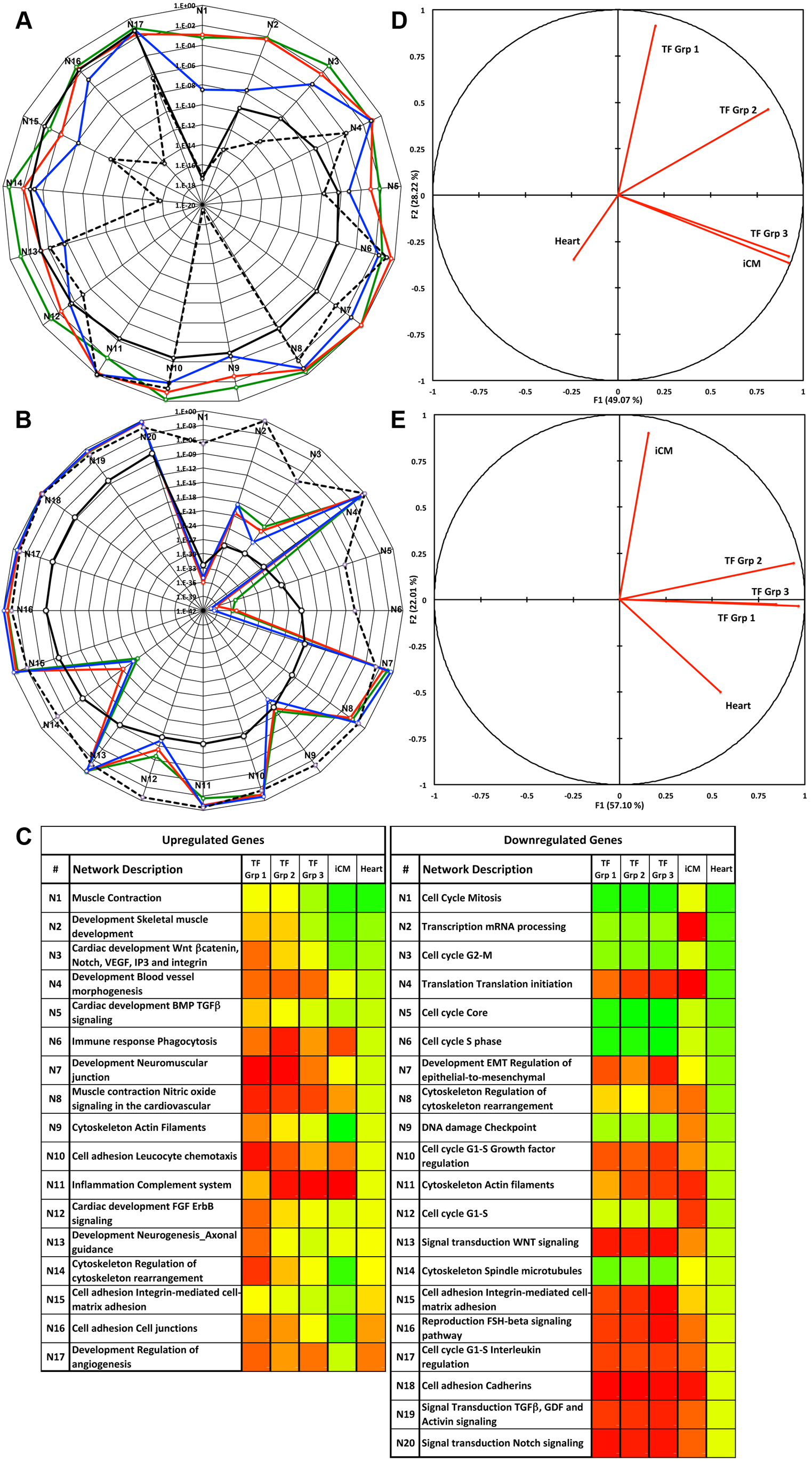

Supplement: Figure S9 — Microarray gene expression analysis performed on populations of transdifferentiated MEFs, Heart Control, and induced cardiomyocytes (iCMs). A–C. Gene process networks that are either activated (A, upregulated genes) or deactivated (B, downregulated genes) in MEFs transduced with the three transcriptional module combinations as compared to negative control, heart positive control as compared to MEFs negative control, and iCMs as compared to cardiac fibroblasts negative control, were determined using the Thomson Reuters GeneGo MetaCore™ data meta-analysis tool (TF Group 1 Green, TF Group 2 Red, TF Group 3 Blue, Heart Control Black, iCMs Doted line). Based on the list of significantly upregulated or downregulated genes each process network received a p-value indicating the statistical probability that the network is affected in the population of transdifferentiated cells, or heart control. The range of calculated p-values for each process network is graphically represented with a green to red color range (C). Activated networks: Lowest p-value: 4.12×10^−18^ (Green) and highest p-value: 9.81×10^−1^ (Red). Deactivated networks: Lowest p-value: 5.17×10^−41^ and highest p-value: 1 (Red). D–E. Principal component analysis was performed on the p-values calculated for each of the process networks for either significantly upregulated (D), or significantly downregulated genes (E). Correlation circle plots are graphically represented for each of the three transcription factor groups, the iCMs, and the heart. (TIF) [file pone.0063577.s009.tif]
